# Molecular mechanisms of Huanglian jiedu decoction on ulcerative colitis based on network pharmacology and molecular docking

**DOI:** 10.1038/s41598-022-09559-1

**Published:** 2022-04-01

**Authors:** Jing Yang, Chaotao Tang, Ruiri Jin, Bixia Liu, Peng Wang, Youxiang Chen, Chunyan Zeng

**Affiliations:** Department of Gastroenterology, The First Affiliated Hospital of Nanchang University, 17 Yongwaizheng Street, Nanchang, 330006 Jiangxi China

**Keywords:** Cell biology, Drug discovery, Gastroenterology

## Abstract

Huanglian jiedu decoction (HLJDD) is a heat-clearing and detoxifying agent composed of four kinds of Chinese herbal medicine. Previous studies have shown that HLJDD can improve the inflammatory response of ulcerative colitis (UC) and maintain intestinal barrier function. However, its molecular mechanism is not completely clear. In this study, we verified the bioactive components (BCI) and potential targets of HLJDD in the treatment of UC using network pharmacology and molecular docking, and constructed the pharmacological network and PPI network. Then the core genes were enriched by GO and KEGG. Finally, the bioactive components were docked with the key targets to verify the binding ability between them. A total of 54 active components related to UC were identified. Ten genes are very important to the PPI network. Functional analysis showed that these target genes were mainly involved in the regulation of cell response to different stimuli, IL-17 signal pathway and TNF signal pathway. The results of molecular docking showed that the active components of HLJDD had a good binding ability with the Hub gene. This study systematically elucidates the “multi-component, multi-target, multi-pathway” mechanism of anti-UC with HLJDD for the first time, suggesting that HLJDD or its active components may be candidate drugs for the treatment of ulcerative colitis.

## Introduction

Ulcerative colitis (UC) is a chronic idiopathic colonic inflammatory bowel disease characterized by intestinal inflammation, mucosal injury, and fibrosis, which can cause different degrees of superficial mucosal inflammation from the rectum to the oral cavity. The pathological features of UC are inflammatory and ulcerative lesions of mucosa and submucosa^1^. The pathogenesis is multifactorial, involving genetic susceptibility, epithelial barrier defects, immune response disorders and environmental factors^2^. The main manifestations of ulcerative colitis are hematochezia, abdominal pain, and diarrhea. The endoscopic biopsy can confirm the diagnosis. The best goal of UC treatment is a sustained and lasting non-steroid remission period, reducing hospitalization and surgery, and minimizing cancer risk^3,4^.

Clinical treatment of ulcerative colitis is difficult, including 5-aminosalicylic acid, steroids, immunosuppressants and biological agents^5^. However, these drugs have many side effects, including headache, nausea, vomiting, diarrhea, nephrotoxicity, and increased risk of infection, which affect the normal work and life of patients. Even some patients did not respond to these drugs^6^. Therefore, it is particularly urgent to find more reliable and effective therapeutic drugs.

Huanglian jiedu decoction (HLJDD), which is composed of four kinds of Chinese herbal medicines, such as Huanglian (HL), Huangbai (HB), Zhizi (ZZ) and Huangqin (HQ), is a representative medicine for heat-clearing and detoxification of traditional Chinese medicine (TCM). It has been widely used in the treatment of gastrointestinal diseases, sepsis, and Alzheimer’s disease in China^7–10^. In addition, recently, associated pharmacological studies show that Huanglian jiedu decoction could function in type II diabetes, inflammatory disease, and ischemic stroke^11–16^. Huanglian jiedu decoction has significant anti-inflammatory effects. In 1999, studies found that HLJDD can reduce experimental colitis in rats^17^. Subsequently, some studies have confirmed that HLJDD can reduce inflammation and intestinal mucosal injury by down-regulating the expression of JAK2 and STAT3, and alleviating DSS-induced colitis in mice^18^. In addition, it can also repair intestinal barrier damage and alleviate ulcerative colitis induced by DSS in mice by regulating NF-κB and NRF2 signal pathway^19^. Due to the multi-component, multi-pathway and multi-target characteristics of traditional Chinese medicine prescription, the mechanism of HLJDD in the treatment of UC remains to be revealed systematically.

Network pharmacology was first proposed by Andrew Hopkins in 2007^20^. It is a new cross-discipline based on the theory of systems biology to study the mechanism of drug action and design multi-target drug molecules at the system level. TCM network pharmacology is a new research paradigm, which aims to transform traditional Chinese medicine from experience-based medicine to evidence-based medicine. We can analyze the interaction network of “traditional Chinese medicine-compound-protein/gene-disease” from the perspective of system biology, to understand the effect of traditional Chinese medicine on disease. TCM Network Pharmacology has also been successfully used to identify the elucidating mechanism of HLJDD in the treatment of pneumonia^21^. Molecular docking is an established method based on a computer simulation structure, which helps to predict the interaction between molecules and biological targets. Through molecular docking, we can verify the binding ability between active compounds and key targets and improve the accuracy of the target network^22^.

**Figure 1:**
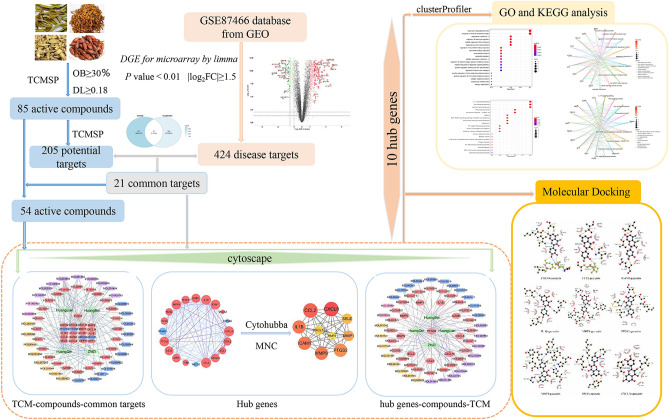
The flowchart of the analysis procedures of the study.

In this study, we used the methods of network pharmacology and molecular docking to study the potential mechanism of HLJDD in the treatment of UC. Firstly, HLJDD bioactive chemical components (BCI) and related target genes were searched and screened in TCMSP, and UC-specific genes were obtained in GEO database. Then, the UC-specific genes related to the BCI of HLJDD were screened by compound-target interaction analysis. Differential expression analysis was used to verify the expression of key target genes in UC and normal samples. Construction of TCM-BCIs-targets pharmacological network and PPI network, screening of top10 gene in PPI network as an important core target of this study. Then the target genes were analyzed by GO and KEGG enrichment analysis. Finally, the binding ability of bioactive components to key targets was verified by molecular docking with AutoDock Tools. The flow chart of the analysis program we studied is as follows (Fig. 1).

## Results

### HLJJD bioactive compounds and related targets

**Figure 2:**
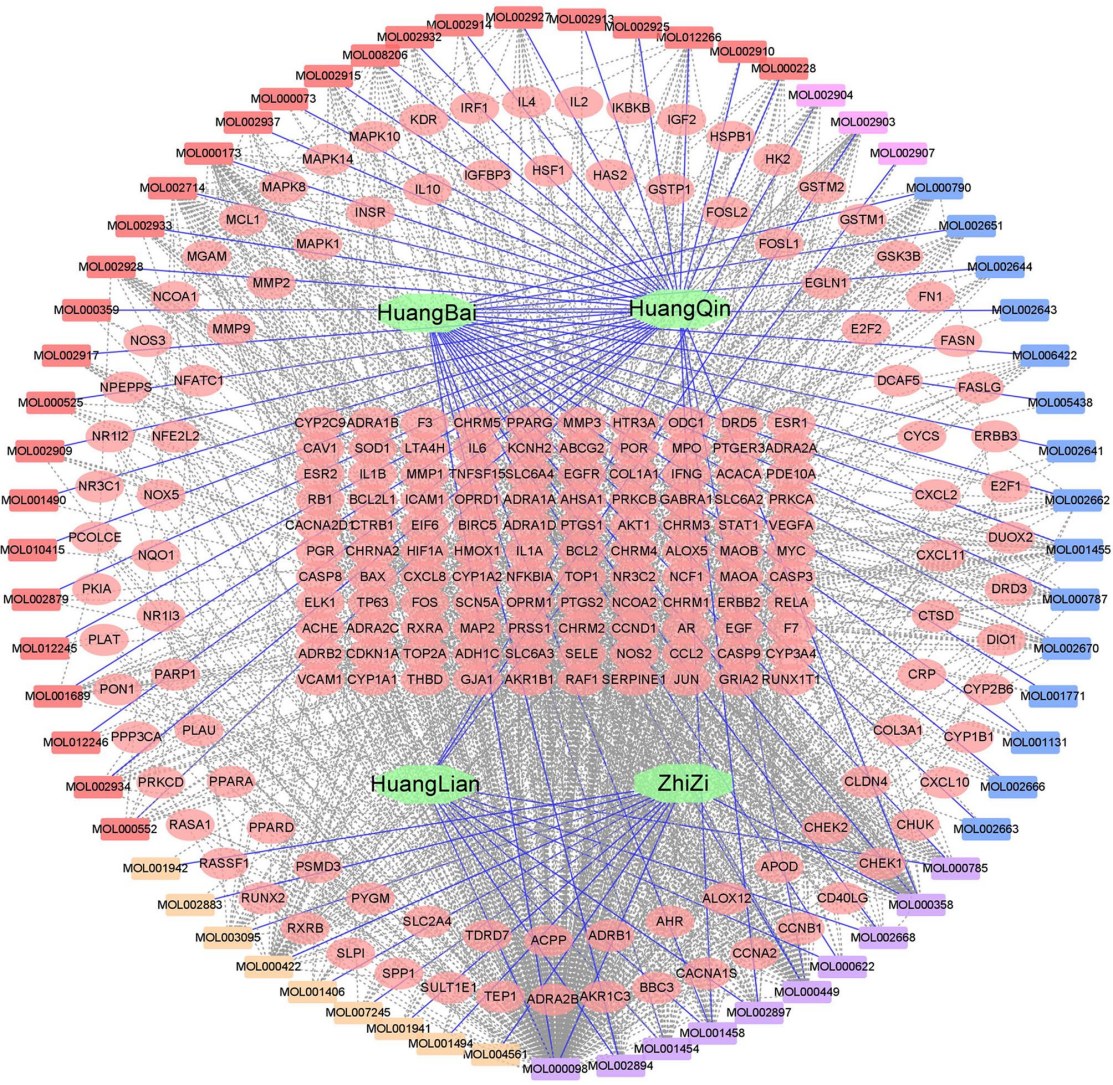
Traditional Chinese medicine-active ingredients-targets network. There are 205 target proteins in the network. Light red circles are used to represent the nodes of the target proteins. The quadrilateral represents the active ingredient, red, yellow, pink and blue represent HQ, ZZ, HL and HB, respectively. The shared BCI of many drugs is painted purple. Traditional Chinese medicine is represented by green hexagons.

429 compounds were searched by TCMSP database: 48 species of HL, 143 species of HQ,140 species of HB and 98 species of ZZ. According to the standards of OB ≥ 30% and DL ≥ 0.18, 102 bioactive components were collected from HLJJD. There are 14 species in HL, 37 species in HB, 36 species in HQ and 15 species in ZZ. The duplicates were excluded and 85 active components were selected for further analysis (as shown in Supplementary Table S1). The corresponding targets of HB, HL, HB and ZZ filtered out by TCMSP were 77, 159, 96 and 171 respectively. After removing the repeated targets, a total of 205 human target proteins were obtained. Draw the traditional Chinese medicine-active ingredients-targets network of HLJJD (Fig. 2). The network consists of 274 nodes (including 205 target nodes, 65 compound nodes and 4 traditional Chinese medicine nodes) and 1337 edges, in which the square node represents the active compound and the traditional Chinese medicine, and the circular node represents the related target. The solid blue lines represent traditional Chinese medicine and the corresponding active ingredients. The gray dotted line represents the interaction between the active compound and the target protein. Among these targets, quercetin (MOL000098), kaempferol (MOL000422) and wogonin (MOL000173) correspond to 50 and 38 targets respectively. Beta-sitosterol (MOL000358) and stigmasterol (MOL000449) correspond to 26 targets. In addition, quercetin, beta-sitosterol and stigmasterol are shared BCI of many drugs. This shows that there are many active ingredients in different drugs, the same ingredients may exist in many kinds of traditional Chinese medicine, and the compounds in the formula of HLJDD may play a role in some common goals, which is an important basis for the multi-component and multi-target action of traditional Chinese medicine, so that HLJDD can play a pharmacological role in UC and other diseases.

### UC specific genes related to BCI of HLJDD

**Figure 3:**
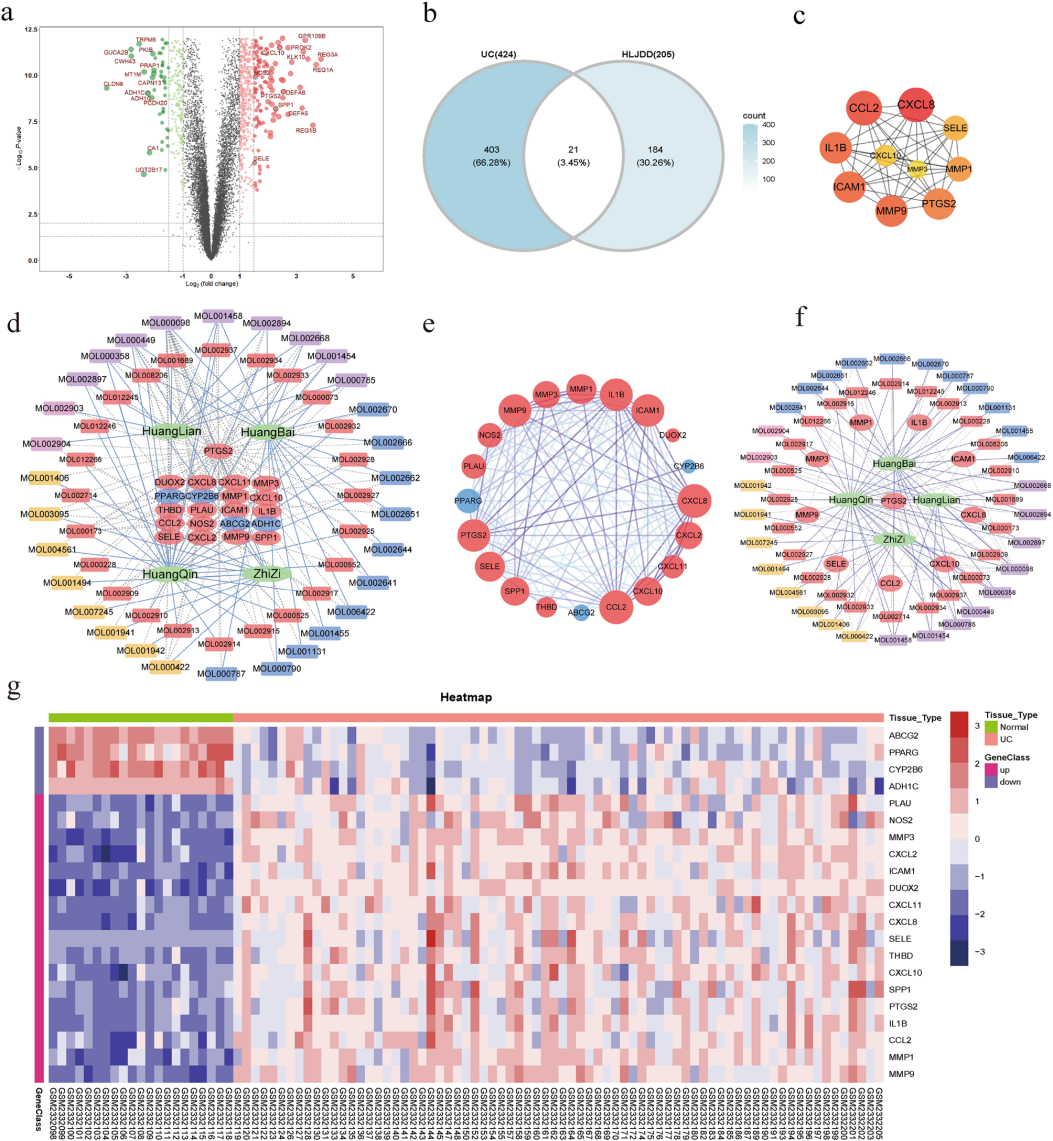
Genetic volcano map, common goal and heat map, TCM-compounds-targets network, PPI network and Hub gene-compounds-TCM network. (**a**) The gene volcano map shows the gene distribution in the disease sample. Red and green represent up-regulated genes (logFC ≥ 1) and down-regulated genes (logFC ≤  − 1) respectively while black indicates no significant difference. The common goal with HLJDD is shown in a black circle. (**b**) The common goal of Huanglian jiedu decoction (HLJDD) and ulcerative colitis (UC). (**c**) PPI networks of Hub gene. The higher the confidence score is, the larger the node size is, and the darker the color will be. (**d**) TCM-compounds-targets network. There are 21 common target proteins in the network, the circle is used to represent the nodes of the target protein, red and blue are used to represent the up-regulated and down-regulated genes in UC tissue, respectively. 54 active components that act on specific genes of UC, which are represented by quadrilaterals. Red, yellow, pink and blue represent HQ, ZZ, HL and HB respectively, and the shared BCI of many drugs is painted purple. Traditional Chinese medicine is represented by green hexagons. The solid blue lines represent traditional Chinese medicine and the corresponding active ingredients. The gray dotted line represents the interaction between the active compound and the target protein. (**e**) 20 common target protein–protein interaction (PPI) networks. The network has 20 nodes and 115 edges. Circles are used to represent the nodes of the target protein, using red and blue to represent up-regulated and down-regulated genes in UC tissue, respectively. The edge is expressed according to the confidence score of the protein–protein interaction relationship, and the higher the score, the darker the color. (**f**) Hub gene-compounds-TCM network. Hub genes, active components, nodes and edge representations are shown in Fig. 3d. PTGS2 is highlighted as the gene with the most active components in the network. (**g**) 21 DEG heat maps, orange and green represent UC samples and normal tissue samples, respectively. Red and purple are used to represent up-regulated and down-regulated genes, respectively. Among them, 17 genes were up-regulated in UC tissues, while the other 4 genes were down-regulated.

In this study, 108 mucosal biopsy samples were included, including 87 ulcerative colitis tissue samples and 21 normal tissue samples. The GSE87466 is analyzed by Rstudio software, and then the DEG set is determined. According to the truncation criteria, a total of 424 genes were identified, of which 279 were up-regulated and 145 were down-regulated (Supplementary Table S2). As shown in the gene volcano map, the differential genes in the disease samples showed normal distribution, and the genes with significant differences were highlighted in the map (Fig. 3a). After crossing 205 BCI-related targets with 424 UC-specific genes, 21 genes were obtained as UC-specific HLJDD target genes (Fig. 3b). Among these genes, MMP3, DUOX2, CXCL8, IL1 β, CXCL2, CXCL11, MMP1, MMP9, PTGS2, CXCL10, SPP1, CCL2, PLAU, ICAM1, THBD, NOS2 and SELE were up-regulated in UC samples, while ABCG2, PPARG, CYP2B6 and ADH1C were down-regulated in UC samples, and the difference was significant (Table 1, Fig. 3g). After excluding the BCI which is not targeted at the specific target of UC, 54 effective BCI are finally used in the construction of a pharmacological network.

**Table 1:** The identified 21 upregulated and downregulated DEGs between UC tissue samples and normal tissue samples.

Gene name	Log_2_FC	P.Value	adj.P.Val	Gene class
ABCG2	− 3.196533333	6.51E−20	1.28E−17	Down
CYP2B6	− 3.014818719	3.67E−20	8.04E−18	Down
ADH1C	− 2.236926765	9.44E−10	1.19E−08	Down
PPARG	− 1.614452709	3.36E−13	1.04E−11	Down
SELE	1.528252874	5.41E−06	2.93E−05	Up
NOS2	1.552404105	1.30E−10	2.02E−09	Up
THBD	1.672966502	3.19E−14	1.25E−12	Up
ICAM1	1.739963711	2.75E−16	1.99E−14	Up
PLAU	1.861776683	1.64E−16	1.23E−14	Up
CCL2	1.952432348	3.36E−13	1.04E−11	Up
SPP1	2.269367816	6.66E−09	6.89E−08	Up
CXCL10	2.408858456	3.19E−12	7.70E−11	Up
PTGS2	2.544686371	7.94E−10	1.02E−08	Up
MMP9	2.56551133	1.32E−17	1.40E−15	Up
MMP1	2.844199507	2.94E−18	3.68E−16	Up
CXCL11	2.912752381	2.18E−14	9.08E−13	Up
CXCL2	3.032518719	7.76E−20	1.50E−17	Up
IL1B	3.052697373	5.21E−16	3.48E−14	Up
CXCL8	3.997391954	2.45E−17	2.40E−15	Up
DUOX2	4.914188342	2.34E−33	1.55E−29	Up
MMP3	5.105582923	2.66E−23	1.18E−20	Up

### Construction of TCM-BCIs-targets pharmacological network, PPI network, and screening of core targets

By comparing the targets of HLJDD and UC, we get a total of 21 targets of HLJDD and UC. According to the common targets, 54 kinds of active components were found (Table 2). The TCM-compounds-common targets network was constructed by Cytoscape software (Fig. 3d). The network consists of 79 nodes (including 21 target nodes, 54 compound nodes and 4 traditional Chinese medicine nodes) and 181 edges, in which the square node represents the active compound and the traditional Chinese medicine, and the circular node represents the related target. Four key active components with degrees ≥ 6 were identified as quercetin (MOL000098), wogonin (MOL000173), kaempferol (MOL000422) and stigmasterol (MOL000449).

**Table 2:** Common targets related to active ingredients.

Mol ID	Molecule name	Target	Source
MOL000098	Quercetin	ABCG2, PPARG, PLAU, MMP3, CXCL2, ICAM1, DUOX2, CXCL11, CXCL8, SELE, THBD, CXCL10, SPP1, PTGS2, IL1B, CCL2, MMP1, MMP9	HuangBai, HuangLian, ZhiZi
MOL000173	Wogonin	MMP9, PPARG, NOS2, CXCL8, PTGS2CCL2, MMP1	HuangQin
MOL000422	Kaempferol	PPARG, NOS2, ICAM1, SELE, PTGS2	ZhiZi
MOL000525	Norwogonin	PPARG, NOS2, PTGS2	HuangQin
MOL002933	5,7,4ʹ-Trihydroxy-8-methoxyflavone	PPARG, NOS2, PTGS2	HuangQin
MOL002934	Neobaicalein	PPARG, NOS2, PTGS2	HuangQin
MOL003095	5-Hydroxy-7-methoxy-2-(3,4,5-trimethoxyphenyl) chromone	PPARG, NOS2, PTGS2	ZhiZi
MOL008206	Moslosooflavone	PPARG, NOS2, PTGS2	HuangQin
MOL000449	Stigmasterol	ADH1C, PLAU, PTGS2	HuangBai, HuangQin, ZhiZi
MOL002662	Rutaecarpine	CYP2B6, PTGS2	HuangBai
MOL000552	5,2ʹ-Dihydroxy-6,7,8-trimethoxyflavone	NOS2, PTGS2	HuangQin
MOL000785	Palmatine	NOS2, PTGS2	HuangBai,HuangLian
MOL001454	Berberine	NOS2, PTGS2	HuangBai,HuangLian
MOL001458	Coptisine	NOS2, PTGS2	HuangBai,HuangLian,HuangQin
MOL001689	Acacetin	NOS2, PTGS2	HuangQin
MOL002668	Worenine	NOS2, PTGS2	HuangBai,HuangLian
MOL002894	Berberrubine	NOS2, PTGS2	HuangBai,HuangLian
MOL002897	Epiberberine	NOS2, PTGS2	HuangLian,HuangQin
MOL002904	Berlambine	NOS2, PTGS2	HuangLian
MOL002909	5,7,2,5-Tetrahydroxy-8,6-dimethoxyflavone	NOS2, PTGS2	HuangQin
MOL002915	Salvigenin	NOS2, PTGS2	HuangQin
MOL002917	5,2ʹ,6ʹ-Trihydroxy-7,8-dimethoxyflavone	NOS2, PTGS2	HuangQin
MOL002927	Skullcapflavone II	NOS2, PTGS2	HuangQin
MOL002928	Oroxylin a	NOS2, PTGS2	HuangQin
MOL002932	Panicolin	NOS2, PTGS2	HuangQin
MOL006422	Thalifendine	NOS2, PTGS2	HuangBai
MOL007245	3-Methylkempferol	NOS2, PTGS2	ZhiZi
MOL012266	Rivularin	NOS2, PTGS2	HuangQin
MOL002651	Dehydrotanshinone II A	PPARG, PTGS2	HuangBai
MOL000073	ent-Epicatechin	PTGS2	HuangQin
MOL000228	(2R)-7-hydroxy-5-methoxy-2-phenylchroman-4-one	PTGS2	HuangQin
MOL000358	Beta-sitosterol	PTGS2	HuangBai,HuangQin,ZhiZi
MOL000787	Fumarine	PTGS2	HuangBai
MOL000790	Isocorypalmine	PTGS2	HuangBai
MOL001131	Phellamurin_qt	PTGS2	HuangBai
MOL001406	Crocetin	PTGS2	ZhiZi
MOL001455	(S)-canadine	PTGS2	HuangBai
MOL001494	Mandenol	PTGS2	ZhiZi
MOL001941	Ammidin	PTGS2	ZhiZi
MOL001942	Isoimperatorin	PTGS2	ZhiZi
MOL002641	Phellavin_qt	PTGS2	HuangBai
MOL002644	Phellopterin	PTGS2	HuangBai
MOL002666	Chelerythrine	PTGS2	HuangBai
MOL002670	Cavidine	PTGS2	HuangBai
MOL002714	Baicalein	PTGS2	HuangQin
MOL002903	(R)-canadine	PTGS2	HuangLian
MOL002910	Carthamidin	PTGS2	HuangQin
MOL002913	Dihydrobaicalin_qt	PTGS2	HuangQin
MOL002914	Eriodyctiol (flavanone)	PTGS2	HuangQin
MOL002925	5,7,2ʹ,6ʹ-Tetrahydroxyflavone	PTGS2	HuangQin
MOL002937	Dihydrooroxylin	PTGS2	HuangQin
MOL004561	Sudan III	PTGS2	ZhiZi
MOL012245	5,7,4ʹ-Trihydroxy-6-methoxyflavanone	PTGS2	HuangQin
MOL012246	5,7,4ʹ-Trihydroxy-8-methoxyflavanone	PTGS2	HuangQin

The protein–protein interaction network (PPI) analysis of the ulcerative colitis target was carried out by using String database online service platform, and all the protein–protein interaction relationships were obtained, and then imported into Cytoscape software for visualization (Fig. 3e). Then the Cytohubba plug-in is used to identify the Hub gene, and the top 10 nodes in the network generated by the MNC method are regarded as the Hub genes: CXCL8, CCL2, ICAM1, IL-1β, MMP9, PTGS2, MMP1, SELE, CXCL10and MMP3 (Fig. 3c). The PPI network with 10 Hub genes has 10 nodes and 45 edges, the average node degree is 9, and the p value is 5.41E-07. The higher the confidence score, the larger the node size and the darker the color. According to the active components related to hub gene, a disease Hub genes-compounds-TCM network was constructed (Supplementary Table S3, Fig. 3f). The most active ingredient of traditional Chinese medicine in the action of HLJDD on UC is HQ, which contains 28 active components that can target UC-specific genes. PTGS2 is targeted by 54 BCI. As an important gene, it is highlighted in the figure that the key active components in the network are quercetin (MOL000098), wogonin (MOL000173), kaempferol (MOL000422).

### GEO and KEGG analysis

**Figure 4:**
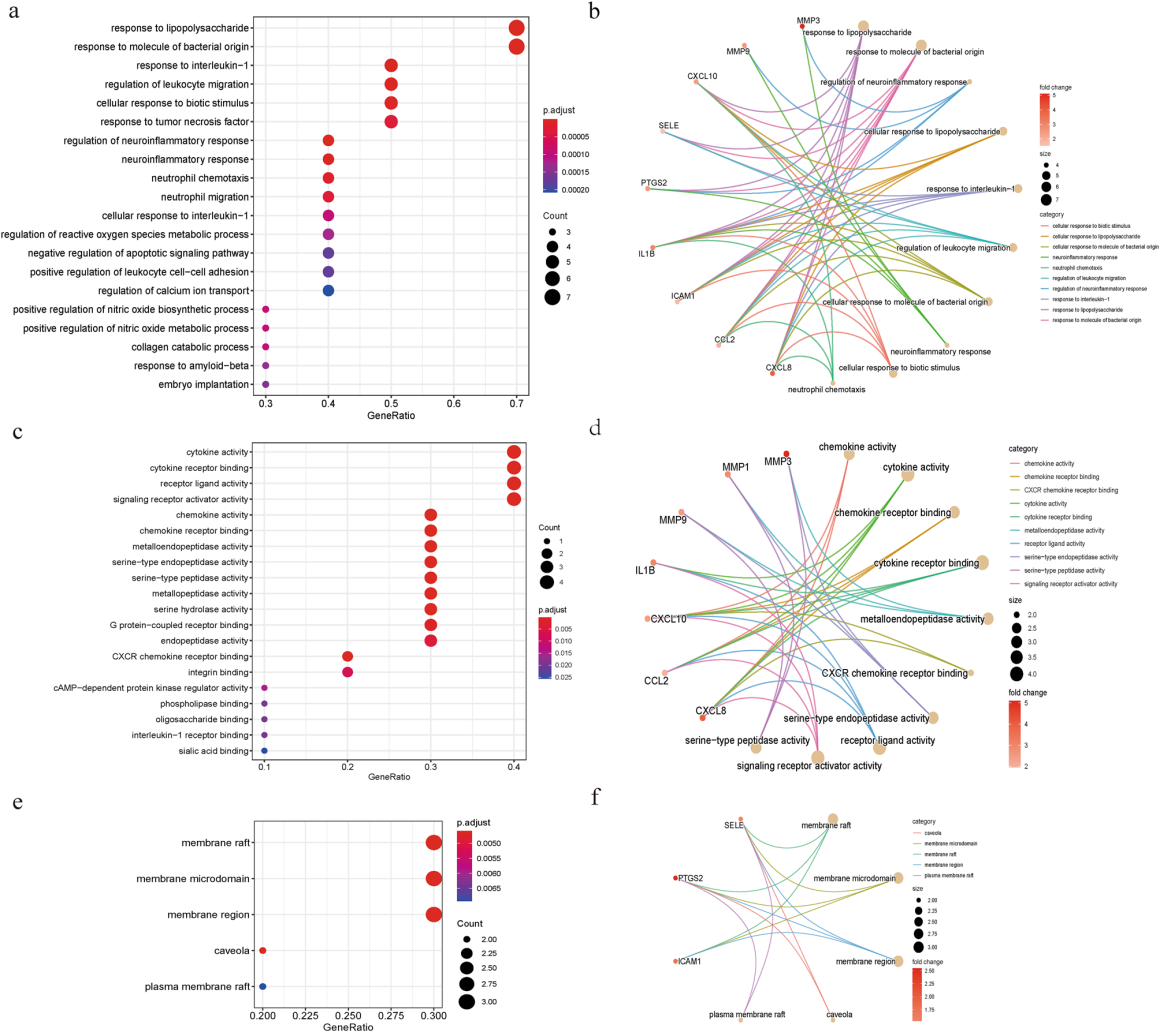
Bubble map of GO-BP, GO-MF, GO-CC and KEGG enrichment analysis and related gene circle map. (**a**) The top 20 noteworthy GO-BP terms enriched by Hub genes in the pharmacological network. The ratio of genes enriched in each term is recorded on the horizontal axis. The gene ratio is the ratio of the number of genes enriched in each term to 10 Hub genes. The larger the bubble, the more genes are involved in the pathway. The color of each bubble represents the adjusted p-value of each GO term. The redder the color of the term, the smaller the adjusted p-value. (**b**) A circle diagram showing the top 10 BP terms and related genes. The line color represents the relevant term. Bubble size represents the number of genes enriched in the term, and the more genes involved, the larger the bubble. Gene color means that the higher the Fold Change, the darker the color. (**c**) The top 20 noteworthy GO-MF terms enriched by Hub genes in the pharmacological network. (**d**) Circle diagram of the top 10 MF terms and related genes. (**e**) The GO-CC terms are enriched by Hub genes in the pharmacological network. (**f**) Circle diagram of 5 CC terms and related genes.

A total of 238 BP terms, 26 MF terms and 5 CC terms were enriched in the 10 target genes (Supplementary Tables S4–S6). The top 20 BP, MF terms and 5 CC terms are shown in a bubble chart (Fig. 4a,c,e). The loop map is used to represent terms and related gene self-networks, showing the related genes (Fig. 4b,d,f) involved in the top 10 BP, MF terms and five CC terms. GO enrichment analysis showed that the key targets were mainly related to cell response to different stimuli, inflammatory response, cell migration and chemokines, such as lipopolysaccharide response (GO:0032496), bacterial molecular response (GO:0002237), regulation of neuroinflammatory response (GO:0150077), regulation of leukocyte migration (GO:0002685) and neutrophil chemotaxis (GO:0030593). The key cellular components are membrane raft (GO:0045121) and membrane microdomain (GO:0098857). The key molecular functions are related to chemokine activity (GO:0008009), cytokine activity (GO:0008009), chemokine receptor binding (GO:0042379), cytokine receptor binding (GO:0005126) and metalloendopeptidase activity (GO:0004222).

A total of 35 pathways were enriched by 10 target genes (Supplementary Table S7). The top 20 KEGG analyses were shown by bubble chart (Supplementary Fig. 1a). Loop maps are used to represent signal pathways and related gene self-networks (Supplementary Fig. 1b). KEGG enrichment analysis showed that the key targets were mainly related to immune response, inflammatory reaction, and disease process. In terms of immune and inflammatory responses, KEGG pathways are classified as IL-17 signaling pathway (hsa04657), TNF signaling pathway (hsa04668) and NF-κB signaling pathway (hsa04064). In terms of involvement in the disease process, the categories of KEGG pathways are lipid and atherosclerosis (hsa05417), rheumatoid arthritis (hsa05323), malaria (hsa05144), age-RAGE signaling pathway in diabetic complications (hsa04933) and coronavirus disease-COVID-19 (hsa05171).

### Molecular docking

**Figure 5:**
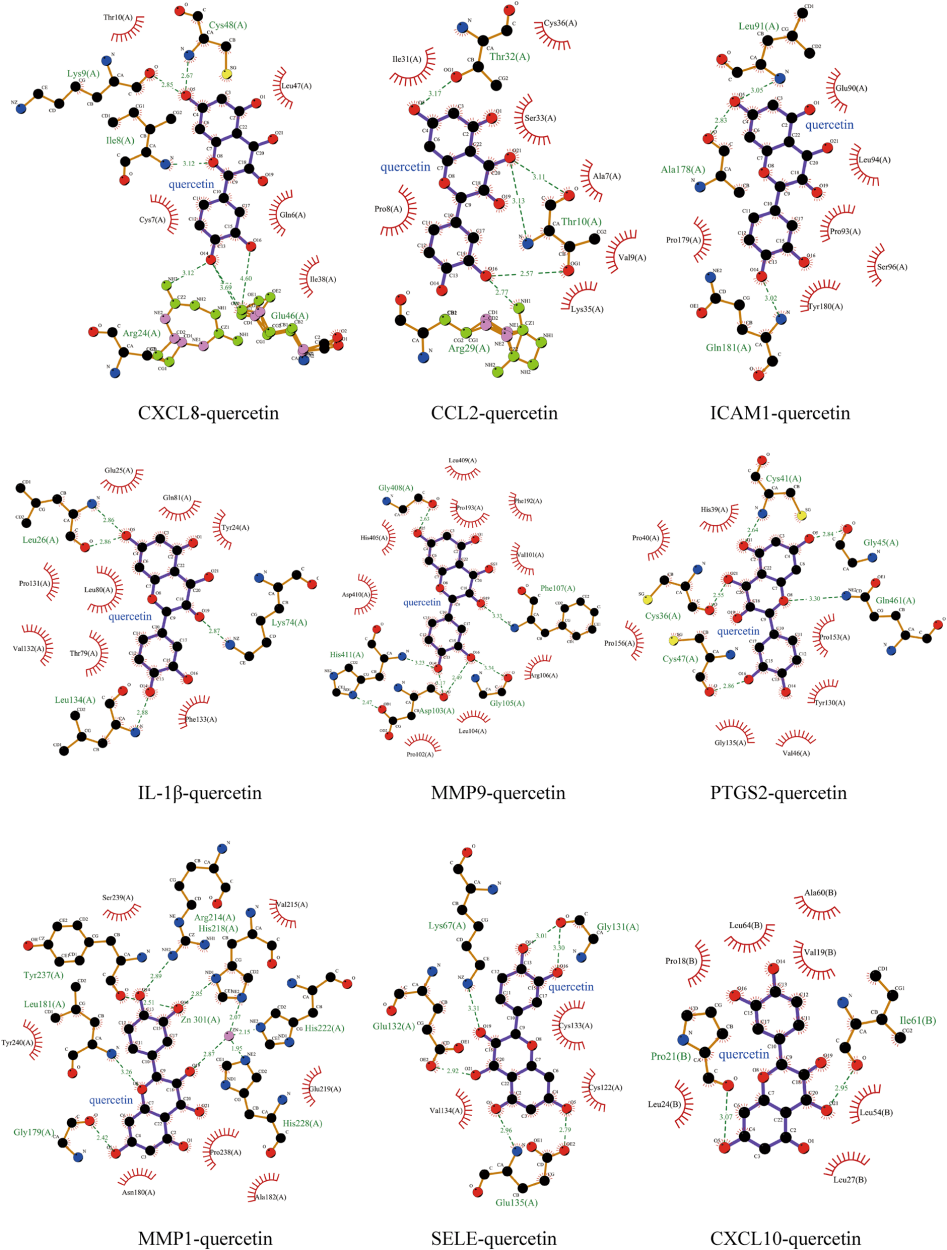
Molecular docking of active ingredients and hub targets. The picture shows the molecular interaction between the protein and the corresponding target, the receptor is highlighted in purple, and the hydrogen bond is shown in green font and the hydrophobic bond is shown in black font.

**Figure 6:**
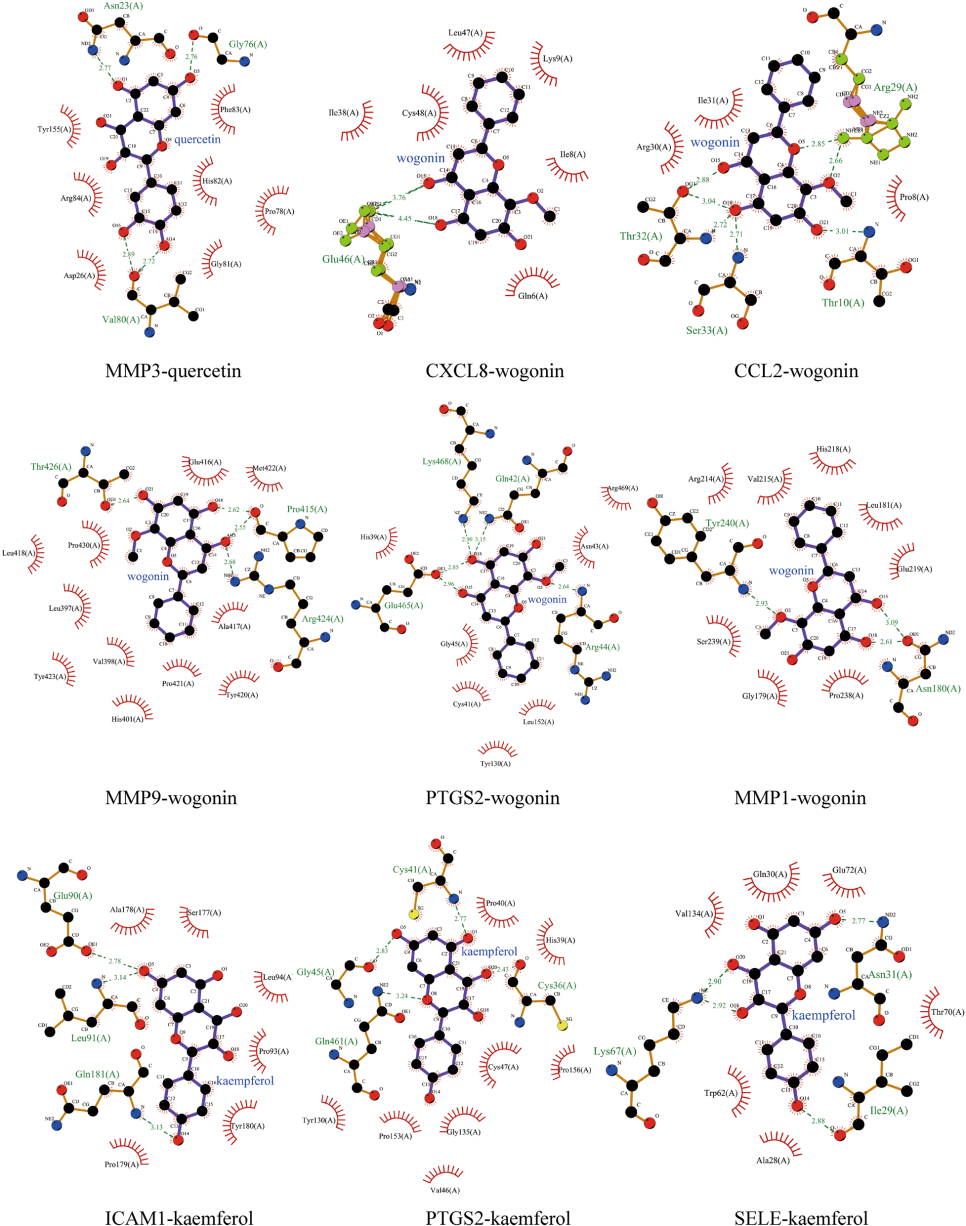
Molecular docking of active ingredients and hub targets. The picture shows the molecular interaction between the protein and the corresponding target, the receptor is highlighted in purple, and the hydrogen bond is shown in green font and the hydrophobic bond is shown in black font.

We use the “analyze network” tool of Cytoscape software to analyze Hub genes-compounds-TCM network. The network consists of 68 nodes and 137 edges. According to the degree of the target corresponding to the active ingredient > 1 as a screening condition, we can observe that except for quercetin, wogonin, kaempferol can target multiple Hub genes, especially quercetin targets all Hub genes, and the other 51 active components target only one of 10 Hub genes: PTGS2. Therefore, quercetin (ZINC3869685), wogonin (ZINC899093) and kaempferol (ZINC3869768) are selected as receptors, and their targeted Hub gene is used as ligands (Fig. 3f, Supplementary Table S3). Then the receptors and ligands are docked to verify their binding activity. The stability of binding between receptors and ligands depends on the binding energy. If the binding energy is less than − 5 kJ/mol, it indicates that the target has a certain binding activity with the compound, and the lower the binding energy, the more stable the binding conformation of the receptor and ligand^23^. The docking results were visualized by LigPlus software (Figs. 5, 6). The results of docking showed that the active component of HLJDD had a good binding ability to the hub gene (Table 3).

**Table 3:** Screening docking results between ligands and receptors.

Active ingredients	Hub targets	Binding energy (kJ/mol)	Hydrophobic interactions	Hydrogen bonds
Quercetin	CXCL8	− 35.44	THR10(A), LEU47(A), CYS7(A), GLN6(A), ILE38(A)	LYS48(A), LYS9(A), ILE8(A), ARG24(A), GLU46A
Quercetin	CCL2	− 25.15	CYS36(A), ILE31(A), SER33(A), PRO8(A), ALA7(A), VAL9(A), LYS35(A)	THR32(A), THR10(A), ARG29(A)
Quercetin	ICAM1	− 23.77	GLU90(A), LEU94(A), PRO179(A), PRO93(A), SER96(A), TYR180(A)	LEU91(A), ALA178(A), GLN181(A)
Quercetin	IL1B	− 34.10	GLU25(A), GLN81(A), TYR24(A), PRO131(A), LEU80(A), VAL132(A), THR79(A), PHE133(A)	LEU26(A), LEU134(A), LYS74(A)
Quercetin	MMP9	− 34.27	LEU409(A), PRO193(A), PHE192(A), HIS405(A), VAL101(A), PRO102(A), ASP410(A), LEU104(A), ARG106(A)	GLY408(A), PHE107(A), HIS411(A), GLY105(A), ASP103(A)
Quercetin	PTGS2	− 30.25	HIS39(A), PRO40(A), PRO156(A), GLY135(A), VAL46(A), TYR130(A), PRO153(A)	CYS41(A), GLY45(A), GLN461(A), CYS36(A), CYS47(A),
Quercetin	MMP3	− 30.75	TYR155(A), PHE83(A), ARG84(A), HIS82(A), PRO78(A), GLY81(A), ASP26(A)	ASN23(A), GLY76(A), VAL80(A)
Quercetin	MMP1	− 31.55	SER239(A), VAL215(A), TYR240(A), ASN180(A), PRO238(A), ALA182(A), GLU219(A)	TYR237(A), LEU181(A), ZN301(A), ARG214(A), HIS218(A), HIS222(A), GLY179(A), HIS228(A)
Quercetin	CXCL10	− 28.66	ALA60(B), LEU64(B), VAL19(B), PRO18(B), LEU24(B), LEU54(B), LEU27(B)	PRO21(B), ILE61(B)
Quercetin	SELE	− 25.86	CYS133(A), CYS122(A), VAL134(A)	LYS67(A), GLY131(A), GLU132(A), GLU135(A)
Wogonin	CXCL8	− 33.56	LEU47(A), LYS9(A), CYS48(A), ILE38(A), ILE8(A), GLN6(A)	GLU46(A)
Wogonin	CCL2	− 26.15	ILE31(A), ARG30(A), PRO8(A)	ARG29(A), THR32(A), SER33(A), THR10(A)
Wogonin	MMP9	− 32.64	GLU416(A), MET422(A), LEU418(A), PRO430(A), LEU397(A), ALA417(A), TYR423(A), HIS401(A), PRO421(A), TYR420(A), VAL398(A)	THR426(A), PRO415(A), ARG424(A)
Wogonin	PTGS2	− 35.15	HIS39(A), ARG469(A), ASN43(A), GLY45(A), CYS41(A), LEU152(A), TYR130(A)	LYS468(A), GLN42(A), GLU465(A), ARG44(A)
Wogonin	MMP1	− 30.12	ARG214(A), VAL215(A), HIS218(A), LEU181(A), GLU219(A), SER239(A), GLY179(A), PRO238(A)	TYR240(A), ASN180(A)
Kaempferol	ICAM1	− 26.19	ALA178(A), SER177(A), LEU94(A), PRO93(A), TYR180(A), PRO179(A)	GLU90(A), LEU91(A), GLN181(A)
Kaempferol	PTGS2	− 30.84	PRO40(A), HIS39(A), CYS47(A), PRO156(A), GLY135(A), PRO153(A), TYR130(A), VAL46(A)	CYS41(A), GLY45(A), GLN461(A), CYS36(A)
Kaempferol	SELE	− 22.97	VAL134(A), GLN30(A), GLU72(A), THR70(A), TRP62(A), ALA28(A)	LYS67(A), ILE2(A), ASN31(A)

## Discussion

Ulcerative colitis (UC) is a chronic idiopathic colonic inflammatory bowel disease. Because its pathogenesis is multi-factor and has the characteristics of recurrence and remission, it makes the treatment of ulcerative colitis more difficult. Clinical treatment is mainly drug therapy, including 5-aminosalicylic acid, steroids, immunosuppressants and biological agents. However, the unnecessary side effects of these drugs affect the normal work and life of patients. Even some patients do not respond to these drugs. Due to the characteristics of natural sources, multi-components, multi-pathways and multi-targets, traditional Chinese medicine prescription has advantages in the treatment of complex diseases, especially in the treatment of diseases with poor response to western medicine alone. Huanglian jiedu decoction (HLJDD) is composed of HL, HB, ZZ and HQ, which is the representative medicine of heat-clearing and detoxification of traditional Chinese medicine. HLJDD has significant anti-inflammatory effects, and many other scholars’ studies are consistent with our findings as early as 1999, when it was found that HLJDD can reduce experimental colitis in rats^17^. Some studies have also confirmed that HLJDD can improve DSS-induced colitis and enhance intestinal barrier function in mice^18,19^. In our study, we systematically discussed the molecular mechanism of HLJDD in the treatment of UC by using the methods of network pharmacology and molecular docking, to provide a more powerful theoretical basis for clinical treatment.

We collected 85 active components of HLJDD through TCMSP database, of which 54 were related to UC targets. Nine BCI are associated with multiple target genes in the pharmacological network. Quercetin (MOL000098), wogonin (MOL000173) and kaempferol (MOL000422) were identified as active components related to most targets. Among the 21 HLJDD targets for UC, 18 targets are involved in quercetin (MOL000098), and kaempferol (MOL000422) and wogonin (MOL000173) correspond to 7 and 5 targets respectively. The results of molecular docking also proved that they have a good binding ability with key target genes. Quercetin is a plant-derived polyphenol compound, and its anti-inflammatory, antioxidant and anti-tumor activities have been reported^24^. Quercetin can reduce the secretion of a variety of inflammatory cytokines in mouse bone marrow-derived dendritic cells (BMDC)^25^. Some studies have shown that quercetin can inhibit LPS-induced inflammation in wild-type organoids and spontaneous inflammation in ulcerative colitis-organoids. Organoids were produced from Winnie, a mouse model of ulcerative colitis^26^. In addition, the anti-inflammatory effect of quercetin is also associated with the decreased expression of Chand EBP-β, a transcriptional factor that can trigger the expression of various inflammatory mediators^27^. Wogonin is a naturally occurring flavonoid, and there has been a lot of evidence that wogonin has the role of anti-inflammatory and antioxidant stress^28,29^. Cartilage protection is achieved by inhibiting molecular events involved in oxidative stress, inflammation and matrix degradation in osteoarthritis chondrocytes and cartilage explants^30^. Wogonin has been shown to inhibit inflammation-related colorectal cancer by inhibiting NF-κB and activating the Nrf2 signaling pathway^31^. Kaempferol is an effective anti-inflammatory agent, and it has been shown that kaempferol can protect mouse colonic mucosa from DSS-induced UC^32^. These findings indicate the potential of HLJDD in the treatment of UC.

Network pharmacological analysis showed that 21 target genes in UC were regulated by HLJDD, including MMP3, DUOX2, CXCL8, IL1 β, CXCL2, CXCL11, MMP1, MMP9, PTGS2, CXCL10, SPP1, CCL2, PLAU, ICAM1, THBD, NOS2, SELE, PPARG, ADH1C, CYP2B6 and ABCG2. We screened the top 10 Hub genes: CXCL8, CCL2, ICAM1, IL-1β, MMP9, PTGS2, MMP1, SELE, CXCL10, MMP3 through the Cytohubba plug-in of Cytoscape software. CXCL8 secreted by immune cells is a chemokine that attracts neutrophils that produce CXCR1 [+] CXCR2 [+] IL-23 to infiltrate and accumulate in inflamed colon tissue^33^. Studies have confirmed that there is excessive secretion of chemokine CXCL8 in colitis and colitis-associated cancer (CAC)^34^. It has been reported that the increase of inflammatory macrophages in IBD patients who did not respond to anti-TNF-α therapy is associated with the upregulation of the TREM-1/CCL7/CCR2 axis^35^. In addition, ICAM1 is a candidate biomarker of UC activity^36^. Matrix metalloproteinases (MMP) can activate cytokines and release isolated growth factors to initiate, amplify or down-regulate signaling cascades involved in growth and inflammation. Overexpression and activation of MMP can cause colonic mucosal injury and inflammation^37^. Studies have shown that MMP1 overexpression is associated with the initial steps of mucosal inflammation and ulcers in UC^38,39^, MMP9 activity is associated with the production and persistence of UC inflammatory state^40,41^, and MMP3 has been shown to play an important role in T cells and TNF-α mediated intestinal injury^42^. Studies have found that IL-1β, MMP9 and CXCL10 may be candidate biomarkers of UC-related cancer^43–45^. In the pharmacological network, PTGS2 (Cyclooxygenase-2, COX-2) is targeted by 54 BCI. Studies have shown that PTGS2 is involved in the development of colitis and CAC^46^. Inhibition of COX-2 can reduce the expression of pro-inflammatory mediators and reduce the symptoms and pathological features of UC in mouse models^47^.

We did KEGG enrichment analysis of these 10 Hub genes, and the results showed that the key targets were mainly related to immune response, inflammatory reaction, and disease process. The therapeutic effect of HLJJD on UC may be achieved by regulating the immune system and inflammation-related pathways. IL-17signalingpathway is one of the most important signal pathways. IL-17 is a T cell-derived cytokine produced by memory CD4+ and CD8+ T cells. The pro-inflammatory property of IL-17 is the key to its host protection ability, but the unrestricted IL-17 signal transduction is related to immunopathology, autoimmune diseases, and cancer progression^48^. IL-17 cytokines play a key role in the pathogenesis of IBD. IL-17 can stimulate the expression of pro-inflammatory cytokines in human cells, and the expression of mucosal IL-17 and serum IL-17 levels in patients with active IBD are increased^49^. In the mouse colitis model induced by acute trinitrobenzene sulfonic acid (TNBS), it was found that IL-17 was produced in 24-h and 48-h colon tissue. IL-17R KO mice showed less severe inflammation in response to acute TNBS treatment and significantly protected against TNBS-induced weight loss^50^. A meta-analysis showed that serum IL-17 levels were significantly correlated with the severity of UC^51^. Tumor necrosis factor (TNF) is a pro-inflammatory mediator that can up-regulate the production of reactive oxygen species (ROS) and reactive nitrogen (RNS) and aggravate cell damage^52^. Proinflammatory cytokine TNF-α plays a key role in coordinating the inflammatory cascade of chronic intestinal inflammation. After binding to the type 1 TNF receptor (TNFR1) and type 2 (TNFR2) receptor, TNF-α activates MAPK and NF-κB pathways and initiates pro-inflammatory signal^53^. MAPK and NF-κB pathways can induce cell proliferation, differentiation, and up-regulation of many proinflammatory cytokines (including TNF-α) during inflammation. In addition, the combination of TNF-α and TNFR1 can also induce intestinal epithelial cell apoptosis^54^. NF-κB is the core regulator of inflammatory response. NF-κB is significantly induced in IBD patients. NF-κB signal pathway can induce the expression of pro-inflammatory cytokines and lead to inflammation-related tissue damage^55,56^. Some studies have shown that both classical and non-classical NF-κB signal transduction is involved in the development of CAC^57,58^.

The studies of many other scholars are consistent with our findings. HLJDD can enhance intestinal barrier function by inhibiting the NF-κB signal pathway and effectively alleviate DSS-induced UC in mice^19^. In addition, HLJDD significantly decreased the disease activity index and inhibited the shortening of colon length and pathological damage in UC mice^59^. At the same time, it can restore the intestinal flora homeostasis of UC mice by inhibiting the growth of intestinal pathogens and preventing the decrease of beneficial bacteria^60^.

This study shows that quercetin, wogonin and kaempferol are the main active ingredients of HLJDD in the treatment of UC. CXCL8, CCL2, ICAM1, IL-1β, MMP9, PTGS2, MMP1, SELE, CXCL10 and MMP3 may be potential therapeutic targets of HLJDD in UC. The therapeutic effect of HLJJD on UC may be achieved by regulating the immune system and inflammation-related pathways. The method of network pharmacology can provide a new and systematic analysis method for the research of Chinese herbal medicine. But our research also has limitations. Focusing on validated targets may rule out some unverified potential targets, and our research needs to be verified by further clinical and basic research.

## Materials and methods

### Acquisition and screening of HLJDD bioactive compounds

Clinical drug treatment is usually oral administration. Human oral bioavailability (OB) ≥ 30% of the compounds have good absorption and slow metabolism after oral administration. The compounds with drug-likeness (DL) ≥ 0.18 are chemically suitable for drug development. By using “Huanglian”, “Huangbai”, “Huangqin” and “Zhizi” as keywords, we screened the bioactive compounds of HLJDD in TCMSP (https://old.tcmsp-e.com/tcmsp.php). According to the drug screening criteria recommended by the TCMSP database, if OB ≥ 30% and DL ≥ 0.18, BCIs retained^21^.

### Targets related to bioactive compounds

According to the active component of HLJDD obtained by TCMSP, the target corresponding to this active component was searched. Using the UniProt database (https://www.uniprot.org/), all target proteins were converted into corresponding gene symbols of the “Homo sapiens” species, and the corresponding target information was obtained^61^.

### The specific gene of UC

Use the following search words: (“colitis, ulcerative” [MeSH Terms] OR ulcerative colitis [All Fields]) AND “Homo sapiens” [porgn], after careful filtering from the GEO (http://www.ncbi.nlm.nih.gov/geo/) database, use the microarray data GSE87466 submitted by Li K et al. This data is based on GPL13158 [HT_HG-U133_Plus_PM] Affymetrix HT HG-U133 + PM Array Plate. The data set included 108 mucosal biopsy samples, including 87 ulcerative colitis tissue samples and 21 normal tissue samples^62^. First of all, the Affy package in R studio software (version 4.0.5) is used to preprocess the original data^63^. Then, the background adjustment, standardization and logarithmic conversion of the expressed data are carried out by using the robust multiarray average (RMA) algorithm^64^. Paired t-test based on microarray data linear model (LIMMA) packet in R was used to identify differentially expressed genes (DEG) between UC and normal samples. P-value criteria < 0.01 and a | log2 fold-change (log2FC) |≥ 1.5 were used as truncation criteria for follow-up analysis.

### UC specific genes related to BCI of HLJDD

According to the related targets of active components of HLJDD obtained by TCMSP and UC specific genes, the genes that are not only HLJDD related targets but also UC specific genes are obtained. Then differential expression analysis was used to verify the expression of key target genes in UC and normal samples.

### Network construction

#### The construction of the pharmacological network

The corresponding active components are found according to the four traditional Chinese medicines of HLJDD, and these components are related to specific genes of UC. UC-specific genes were obtained by GEO database. A TCM-compounds-targets network was constructed using Cytoscape software (version3.8.2) to illustrate the anti-UC regulation mechanism between the BCI of HLJDD and their specific targets^65^.

#### Construction of protein–protein interaction (PPI) network and screening of core targets

Import HLJDD BCI-related UC-specific targets into the String database (https://string-db.org/). The species was set as “Homo sapiens”, and all protein–protein interaction relationships were obtained, and the confidence score was ≥ 0.4^66^. Then it was imported into Cytoscape software to generate the PPI network diagram of UC regulated by the active components of Huanglian jiedu decoction. The Cytohubba plug-in was used to identify the hub gene, and the top 10 genes generated by the maximum neighborhood component (MNC) method were regarded as the hub gene^21,67^. For each node in an interactive network, the degree represents the number of edges between one node and other nodes in the network, which measures the number of connections to other nodes and reflects the importance of the node^68^.

#### Construction of disease core target-active ingredients-traditional Chinese medicine network

According to the active components related to the hub gene, a Hub targets-compounds-TCM network was constructed. HLJDD acts on the most active traditional Chinese medicine and active components of the core genes of the disease.

### Gene Ontology (GO) functional annotation and Kyoto encyclopedia of genes and genomes (KEGG) pathway analysis

The hub gene selected by the MNC method was analyzed by GO functional enrichment analysis, that is, biological process (BPs), cellular component (CCs) and molecular function (MFs). To clarify the role of target proteins in signal transduction, KEGG pathway analysis was carried out. The enrichment analysis was carried out through the clusterProfiler software package of the R platform, and corrected by the Bonferroni method, the adjusted GO term and adjusted KEGG pathway were of great significance^69–72^.

### Compounds-targets molecular docking

Semi-flexible docking was carried out by AutoDock Tools (version4.2.6) to verify the binding ability of bioactive components to key targets^73,74^. The specific operations are as follows: (1) preparation of receptor molecules: the 3D structures of 10 Hub gene receptors were obtained from the RCSBPDB database (https://www.rcsb.org/) and saved in PDB format (Supplementary Table S8). Then the ligand small molecules and water molecules of the protein were removed by PyMOL software (Version2.4.0), and the PDB format was saved. (2) Preparation of ligand small molecules: Download the 3D structure file of active ingredients from the ZINC (http://zinc.docking.org/) website, and export it to mol2 format. (3) *Molecular docking* Hydrogen and Gasteiger charges were added to the above receptors and ligands by AutoDock Tools (ADT) and stored in PDBQT format. Then use the AutoGrid Tool to set the docking frame parameters, and the grid box contains the entire receptor. We chose the Lamarckian genetic algorithm (LGA) to blind dock the active components with their respective protein targets to generate 100 binding conformations and record the docking sites and binding energies of receptors and ligands when the binding energy was the lowest. Finally, the docking results are visualized by LigPlus software.

## Supplementary Information

Supplementary Information.
