# Supplementary material for: Molecular mechanisms of Huanglian jiedu decoction on ulcerative colitis based on network pharmacology and molecular docking
Source: Sci Rep. 2022 Apr 1;12:5526. doi: 10.1038/s41598-022-09559-1 (PMC8972650; doi:10.1038/s41598-022-09559-1)
Supplement: Supplementary file 1 — Supplementary Information. [file 41598_2022_9559_MOESM1_ESM.docx]

Supplementary Figure 1: KEGG enrichment analysis and related gene circle map.


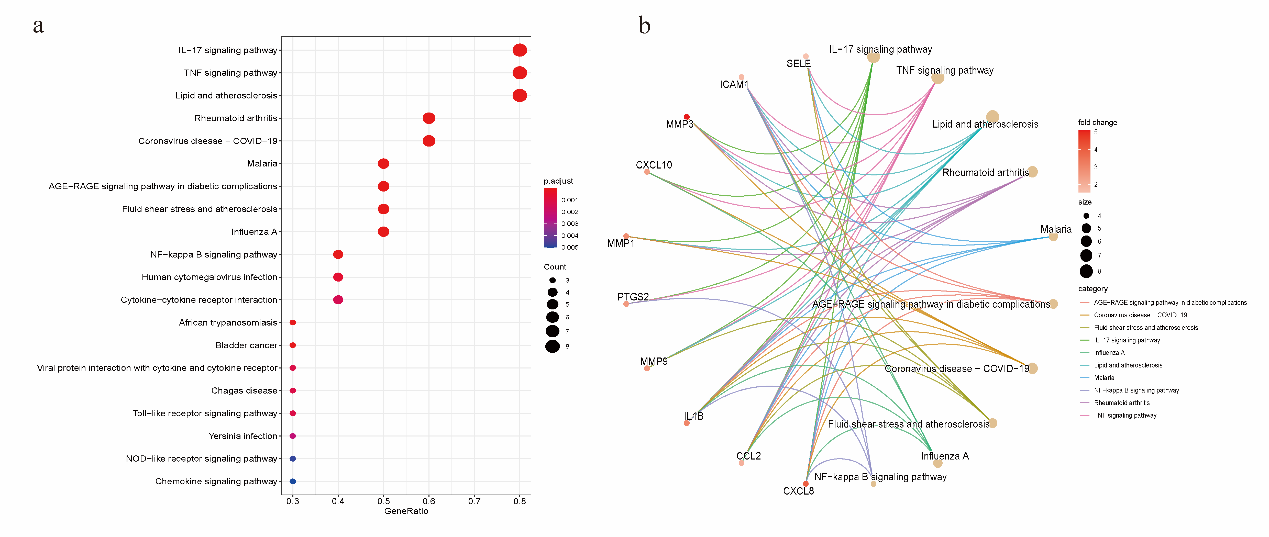


(a) The top 20 KEGG pathways are enriched by the Hub gene. The ratio of genes enriched in each pathway is recorded on the horizontal axis. The size of each bubble indicates the number of genes enriched in each KEGG pathway. The larger the bubble, the more genes are involved in the pathway. The color of each bubble represents the adjusted p-value of each KEGG path. The redder the color of the term, the smaller the adjusted p-value. (b)The circle diagram of the top 10 KEGG pathways and related genes. The line color represents the relevant path. Bubble size represents the number of genes enriched in the pathway, and the more genes involved, the larger the bubble. Gene color means that the higher the Fold Change, the darker the color.

Supplementary Table S1: Information for 85 BCIs of HLJDD.

| Mol ID | Molecule Name | OB (%) | DL | Source |
| --- | --- | --- | --- | --- |
| MOL000073 | ent-Epicatechin | 48.96 | 0.24 | HuangQin |
| MOL000098 | quercetin | 46.43 | 0.28 | HuangBai,HuangLianZhiZi |
| MOL000173 | wogonin | 30.68 | 0.23 | HuangQin |
| MOL000228 | (2R)-7-hydroxy-5-methoxy-2-phenylchroman-4-one | 55.23 | 0.2 | HuangQin |
| MOL000358 | beta-sitosterol | 36.91 | 0.75 | HuangBai,HuangQin,ZhiZi |
| MOL000359 | sitosterol | 36.91 | 0.75 | HuangQin |
| MOL000422 | kaempferol | 41.88 | 0.24 | ZhiZi |
| MOL000449 | Stigmasterol | 43.83 | 0.76 | HuangBai,HuangQin,ZhiZi |
| MOL000525 | Norwogonin | 39.4 | 0.21 | HuangQin |
| MOL000552 | 5,2'-Dihydroxy-6,7,8-trimethoxyflavone | 31.71 | 0.35 | HuangQin |
| MOL000622 | Magnograndiolide | 63.71 | 0.19 | HuangBai,HuangLian |
| MOL000762 | Palmidin A | 35.36 | 0.65 | HuangBai,HuangLian |
| MOL000785 | palmatine | 64.6 | 0.65 | HuangBai,HuangLian |
| MOL000787 | Fumarine | 59.26 | 0.83 | HuangBai |
| MOL000790 | Isocorypalmine | 35.77 | 0.59 | HuangBai |
| MOL001131 | phellamurin_qt | 56.6 | 0.39 | HuangBai |
| MOL001406 | crocetin | 35.3 | 0.26 | ZhiZi |
| MOL001454 | berberine | 36.86 | 0.78 | HuangBai,HuangLian |
| MOL001455 | (S)-Canadine | 53.83 | 0.77 | HuangBai |
| MOL001458 | coptisine | 30.67 | 0.86 | HuangBai,HuangLianHuangQin |
| MOL001490 | bis[(2S)-2-ethylhexyl] benzene-1,2-dicarboxylate | 43.59 | 0.35 | HuangQin |
| MOL001494 | Mandenol | 42 | 0.19 | ZhiZi |
| MOL001506 | Supraene | 33.55 | 0.42 | HuangQin,ZhiZi |
| MOL001663 | (4aS,6aR,6aS,6bR,8aR,10R,12aR,14bS)-10-hydroxy-2,2,6a,6b,9,9,12a-heptamethyl-1,3,4,5,6,6a,7,8,8a,10,11,12,13,14b-tetradecahydropicene-4a-carboxylic acid | 32.03 | 0.76 | ZhiZi |
| MOL001689 | acacetin | 34.97 | 0.24 | HuangQin |
| MOL001771 | poriferast-5-en-3beta-ol | 36.91 | 0.75 | HuangBai |
| MOL001941 | Ammidin | 34.55 | 0.22 | ZhiZi |
| MOL001942 | isoimperatorin | 45.46 | 0.23 | ZhiZi |
| MOL002636 | Kihadalactone A | 34.21 | 0.82 | HuangBai |
| MOL002641 | Phellavin_qt | 35.86 | 0.44 | HuangBai |
| MOL002643 | delta 7-stigmastenol | 37.42 | 0.75 | HuangBai |
| MOL002644 | Phellopterin | 40.19 | 0.28 | HuangBai |
| MOL002651 | Dehydrotanshinone II A | 43.76 | 0.4 | HuangBai |
| MOL002652 | delta7-Dehydrosophoramine | 54.45 | 0.25 | HuangBai |
| MOL002656 | dihydroniloticin | 36.43 | 0.81 | HuangBai |
| MOL002659 | kihadanin A | 31.6 | 0.7 | HuangBai |
| MOL002660 | niloticin | 41.41 | 0.82 | HuangBai |
| MOL002662 | rutaecarpine | 40.3 | 0.6 | HuangBai |
| MOL002663 | Skimmianin | 40.14 | 0.2 | HuangBai |
| MOL002666 | Chelerythrine | 34.18 | 0.78 | HuangBai |
| MOL002668 | Worenine | 45.83 | 0.87 | HuangBai,HuangLian |
| MOL002670 | Cavidine | 35.64 | 0.81 | HuangBai |
| MOL002671 | Candletoxin A | 31.81 | 0.69 | HuangBai |
| MOL002672 | Hericenone H | 39 | 0.63 | HuangBai |
| MOL002673 | Hispidone | 36.18 | 0.83 | HuangBai |
| MOL002714 | baicalein | 33.52 | 0.21 | HuangQin |
| MOL002879 | Diop | 43.59 | 0.39 | HuangQin |
| MOL002883 | Ethyl oleate (NF) | 32.4 | 0.19 | ZhiZi |
| MOL002894 | berberrubine | 35.74 | 0.73 | HuangBai,HuangLian |
| MOL002897 | epiberberine | 43.09 | 0.78 | HuangLian,HuangQin |
| MOL002903 | (R)-Canadine | 55.37 | 0.77 | HuangLian |
| MOL002904 | Berlambine | 36.68 | 0.82 | HuangLian |
| MOL002907 | Corchoroside A_qt | 104.95 | 0.78 | HuangLian |
| MOL002908 | 5,8,2'-Trihydroxy-7-methoxyflavone | 37.01 | 0.27 | HuangQin |
| MOL002909 | 5,7,2,5-tetrahydroxy-8,6-dimethoxyflavone | 33.82 | 0.45 | HuangQin |
| MOL002910 | Carthamidin | 41.15 | 0.24 | HuangQin |
| MOL002911 | 2,6,2',4'-tetrahydroxy-6'-methoxychaleone | 69.04 | 0.22 | HuangQin |
| MOL002913 | Dihydrobaicalin_qt | 40.04 | 0.21 | HuangQin |
| MOL002914 | Eriodyctiol (flavanone) | 41.35 | 0.24 | HuangQin |
| MOL002915 | Salvigenin | 49.07 | 0.33 | HuangQin |
| MOL002917 | 5,2',6'-Trihydroxy-7,8-dimethoxyflavone | 45.05 | 0.33 | HuangQin |
| MOL002925 | 5,7,2',6'-Tetrahydroxyflavone | 37.01 | 0.24 | HuangQin |
| MOL002926 | dihydrooroxylin A | 38.72 | 0.23 | HuangQin |
| MOL002927 | Skullcapflavone II | 69.51 | 0.44 | HuangQin |
| MOL002928 | oroxylin a | 41.37 | 0.23 | HuangQin |
| MOL002932 | Panicolin | 76.26 | 0.29 | HuangQin |
| MOL002933 | 5,7,4'-Trihydroxy-8-methoxyflavone | 36.56 | 0.27 | HuangQin |
| MOL002934 | NEOBAICALEIN | 104.34 | 0.44 | HuangQin |
| MOL002937 | DIHYDROOROXYLIN | 66.06 | 0.23 | HuangQin |
| MOL003095 | 5-hydroxy-7-methoxy-2-(3,4,5-trimethoxyphenyl) chromone | 51.96 | 0.41 | ZhiZi |
| MOL004561 | Sudan III | 84.07 | 0.59 | ZhiZi |
| MOL005438 | campesterol | 37.58 | 0.71 | HuangBai |
| MOL006392 | dihydroniloticin | 36.43 | 0.82 | HuangBai |
| MOL006401 | melianone | 40.53 | 0.78 | HuangBai |
| MOL006413 | phellochin | 35.41 | 0.82 | HuangBai |
| MOL006422 | thalifendine | 44.41 | 0.73 | HuangBai |
| MOL007245 | 3-Methylkempferol | 60.16 | 0.26 | ZhiZi |
| MOL008206 | Moslosooflavone | 44.09 | 0.25 | HuangQin |
| MOL008647 | Moupinamide | 86.71 | 0.26 | HuangLian |
| MOL009038 | GBGB | 45.58 | 0.83 | ZhiZi |
| MOL010415 | 11,13-Eicosadienoic acid, methyl ester | 39.28 | 0.23 | HuangQin |
| MOL012245 | 5,7,4'-trihydroxy-6-methoxyflavanone | 36.63 | 0.27 | HuangQin |
| MOL012246 | 5,7,4'-trihydroxy-8-methoxyflavanone | 74.24 | 0.26 | HuangQin |
| MOL012266 | rivularin | 37.94 | 0.37 | HuangQin |
| MOL013352 | Obacunone | 43.29 | 0.77 | HuangBai,HuangLian |

Supplementary Table S2: The DEGs between UC tissue samples and normal tissue samples.

| Gene name | Log_2_FC | P.Value | adj.P.Val | Gene class |
| --- | --- | --- | --- | --- |
| MMP3 | 5.105582923 | 2.66E-23 | 1.18E-20 | up |
| SLC6A14 | 5.009476355 | 3.25E-41 | 6.46E-37 | up |
| DUOX2 | 4.914188342 | 2.34E-33 | 1.55E-29 | up |
| DEFB4A | 4.583657307 | 1.28E-14 | 5.71E-13 | up |
| CHI3L1 | 4.566604926 | 3.25E-22 | 1.06E-19 | up |
| S100A8 | 4.311166831 | 2.43E-22 | 8.35E-20 | up |
| IL8 | 3.997391954 | 2.45E-17 | 2.40E-15 | up |
| CXCL8 | 3.997391954 | 2.45E-17 | 2.40E-15 | up |
| TNIP3 | 3.882058292 | 3.68E-19 | 6.01E-17 | up |
| REG3A | 3.873189491 | 1.36E-11 | 2.71E-10 | up |
| CXCL1 | 3.814936782 | 1.12E-27 | 1.71E-24 | up |
| REG1A | 3.706336289 | 2.70E-11 | 5.01E-10 | up |
| REG1B | 3.579290969 | 5.15E-08 | 4.35E-07 | up |
| VNN1 | 3.417585386 | 2.27E-24 | 1.46E-21 | up |
| MMP10 | 3.413122496 | 5.89E-19 | 8.96E-17 | up |
| KLK10 | 3.410769622 | 1.39E-11 | 2.76E-10 | up |
| FCGR3B | 3.395420033 | 2.72E-15 | 1.43E-13 | up |
| GPR109B | 3.315846141 | 1.32E-12 | 3.47E-11 | up |
| AQP9 | 3.31164532 | 7.76E-13 | 2.16E-11 | up |
| PROK2 | 3.244370936 | 5.52E-12 | 1.23E-10 | up |
| DUOXA2 | 3.242964204 | 1.42E-22 | 5.32E-20 | up |
| DEFA5 | 3.205622167 | 6.18E-09 | 6.45E-08 | up |
| SERPINB5 | 3.203710345 | 7.04E-19 | 1.04E-16 | up |
| TCN1 | 3.168626437 | 6.88E-14 | 2.51E-12 | up |
| DEFA6 | 3.133857964 | 4.65E-10 | 6.32E-09 | up |
| LOC100288985 | 3.071798686 | 9.08E-14 | 3.22E-12 | up |
| PRRX1 | 3.066507718 | 2.37E-23 | 1.09E-20 | up |
| IDO1 | 3.060743514 | 7.02E-16 | 4.49E-14 | up |
| IL1B | 3.052697373 | 5.21E-16 | 3.48E-14 | up |
| CXCL2 | 3.032518719 | 7.76E-20 | 1.50E-17 | up |
| IL1RN | 3.017757964 | 9.17E-26 | 1.07E-22 | up |
| CXCL9 | 2.978891461 | 3.77E-18 | 4.53E-16 | up |
| SOCS3 | 2.960253202 | 9.99E-20 | 1.88E-17 | up |
| SERPINA3 | 2.959840394 | 8.47E-16 | 5.32E-14 | up |
| CXCL11 | 2.912752381 | 2.18E-14 | 9.08E-13 | up |
| TRIM29 | 2.885618555 | 7.34E-25 | 5.85E-22 | up |
| MMP1 | 2.844199507 | 2.94E-18 | 3.68E-16 | up |
| CXCL13 | 2.843093268 | 1.98E-11 | 3.77E-10 | up |
| LCN2 | 2.833235961 | 2.10E-31 | 5.98E-28 | up |
| PDPN | 2.81887931 | 2.73E-21 | 7.60E-19 | up |
| CTLA4 | 2.811913957 | 5.32E-19 | 8.28E-17 | up |
| IL13RA2 | 2.794836453 | 8.52E-11 | 1.38E-09 | up |
| KYNU | 2.768413793 | 2.50E-20 | 5.78E-18 | up |
| CXCL5 | 2.708377833 | 1.31E-08 | 1.27E-07 | up |
| STC1 | 2.707484236 | 3.36E-12 | 8.04E-11 | up |
| TNC | 2.666145484 | 4.73E-17 | 4.34E-15 | up |
| BCL2A1 | 2.649308539 | 2.91E-15 | 1.51E-13 | up |
| CXCL6 | 2.640872414 | 8.46E-14 | 3.02E-12 | up |
| FPR1 | 2.613876026 | 5.12E-14 | 1.93E-12 | up |
| GREM1 | 2.583280296 | 1.99E-15 | 1.11E-13 | up |
| MMP12 | 2.581396552 | 3.81E-20 | 8.11E-18 | up |
| MMP9 | 2.56551133 | 1.32E-17 | 1.40E-15 | up |
| CXCL3 | 2.559807389 | 7.20E-23 | 2.87E-20 | up |
| TDO2 | 2.549479475 | 5.68E-15 | 2.78E-13 | up |
| PTGS2 | 2.544686371 | 7.94E-10 | 1.02E-08 | up |
| KIAA1199 | 2.533436782 | 1.92E-10 | 2.85E-09 | up |
| S100A12 | 2.528511823 | 1.72E-09 | 2.03E-08 | up |
| S100A9 | 2.522174877 | 2.30E-14 | 9.51E-13 | up |
| CCL18 | 2.509685714 | 1.02E-12 | 2.77E-11 | up |
| TNFAIP6 | 2.479783744 | 1.77E-10 | 2.65E-09 | up |
| CDH3 | 2.412320197 | 7.93E-22 | 2.47E-19 | up |
| DUOXA1 | 2.411326929 | 6.81E-18 | 7.88E-16 | up |
| LAMP3 | 2.409577833 | 1.28E-16 | 9.96E-15 | up |
| CXCL10 | 2.408858456 | 3.19E-12 | 7.70E-11 | up |
| C2CD4A | 2.398782594 | 4.54E-19 | 7.35E-17 | up |
| C4orf7 | 2.381729064 | 1.77E-08 | 1.66E-07 | up |
| SELL | 2.360595731 | 1.78E-12 | 4.54E-11 | up |
| DMBT1 | 2.358322824 | 1.59E-14 | 6.91E-13 | up |
| CYR61 | 2.351509524 | 4.81E-17 | 4.40E-15 | up |
| SELP | 2.331929557 | 3.96E-15 | 2.02E-13 | up |
| PDE4B | 2.3227867 | 9.55E-16 | 5.87E-14 | up |
| PI3 | 2.313507389 | 2.66E-27 | 3.78E-24 | up |
| C3 | 2.31234335 | 6.43E-16 | 4.18E-14 | up |
| CD274 | 2.302016256 | 2.96E-16 | 2.11E-14 | up |
| C4BPB | 2.293817734 | 3.76E-17 | 3.56E-15 | up |
| SPP1 | 2.269367816 | 6.66E-09 | 6.89E-08 | up |
| THBS2 | 2.262248933 | 2.39E-11 | 4.50E-10 | up |
| ZBP1 | 2.257488506 | 6.76E-17 | 5.73E-15 | up |
| CLDN2 | 2.257188506 | 1.25E-17 | 1.33E-15 | up |
| MMP7 | 2.223987356 | 3.45E-14 | 1.34E-12 | up |
| LTF | 2.216952381 | 1.31E-08 | 1.27E-07 | up |
| SLC6A20 | 2.194490476 | 2.16E-15 | 1.19E-13 | up |
| MS4A1 | 2.184413136 | 2.65E-08 | 2.40E-07 | up |
| CFB | 2.184179967 | 6.57E-24 | 3.49E-21 | up |
| FGR | 2.182579803 | 1.88E-16 | 1.39E-14 | up |
| KCND3 | 2.161907553 | 2.77E-23 | 1.20E-20 | up |
| EGFL6 | 2.155565189 | 6.55E-10 | 8.62E-09 | up |
| TWIST1 | 2.147639573 | 1.80E-13 | 6.00E-12 | up |
| CHI3L2 | 2.143307225 | 4.56E-11 | 7.96E-10 | up |
| IL24 | 2.142160099 | 3.80E-09 | 4.14E-08 | up |
| NR4A3 | 2.138506732 | 2.10E-10 | 3.07E-09 | up |
| SLC2A3 | 2.129349425 | 1.33E-15 | 7.84E-14 | up |
| CLEC4E | 2.129255172 | 2.42E-12 | 5.99E-11 | up |
| GZMB | 2.123915764 | 4.53E-13 | 1.36E-11 | up |
| TIMP1 | 2.115407718 | 1.94E-32 | 6.42E-29 | up |
| CSF3R | 2.109347455 | 7.46E-11 | 1.23E-09 | up |
| TGM2 | 2.109221182 | 3.46E-22 | 1.09E-19 | up |
| RGS4 | 2.100563218 | 1.42E-10 | 2.18E-09 | up |
| TACSTD2 | 2.098512808 | 1.20E-14 | 5.40E-13 | up |
| PCDH17 | 2.091103777 | 3.99E-16 | 2.78E-14 | up |
| PRAC | 2.090096059 | 2.10E-07 | 1.52E-06 | up |
| SERPINB7 | 2.084840066 | 1.32E-07 | 1.01E-06 | up |
| EMR2 | 2.083907225 | 4.79E-15 | 2.39E-13 | up |
| CXCR2 | 2.074908046 | 4.24E-10 | 5.80E-09 | up |
| REG4 | 2.074883415 | 2.41E-23 | 1.09E-20 | up |
| FAM40B | 2.074322496 | 9.19E-16 | 5.72E-14 | up |
| GZMK | 2.053637603 | 1.15E-16 | 9.21E-15 | up |
| SIK1 | 2.049714614 | 1.51E-16 | 1.15E-14 | up |
| CFI | 2.042413136 | 6.41E-14 | 2.36E-12 | up |
| JAK3 | 2.014273235 | 1.78E-13 | 5.96E-12 | up |
| PLA2G2D | 2.010565681 | 2.78E-09 | 3.13E-08 | up |
| S100A7 | 1.996610016 | 8.80E-06 | 4.53E-05 | up |
| NNMT | 1.995039573 | 1.22E-18 | 1.65E-16 | up |
| FAP | 1.993049589 | 2.63E-10 | 3.78E-09 | up |
| CCL20 | 1.977201642 | 3.32E-11 | 6.02E-10 | up |
| PDZK1IP1 | 1.967794417 | 3.62E-21 | 9.49E-19 | up |
| MNDA | 1.966669294 | 3.79E-12 | 8.94E-11 | up |
| FIBIN | 1.959890969 | 2.53E-11 | 4.72E-10 | up |
| CD55 | 1.957441215 | 5.16E-20 | 1.07E-17 | up |
| OSMR | 1.953289327 | 2.03E-18 | 2.60E-16 | up |
| CCL2 | 1.952432348 | 3.36E-13 | 1.04E-11 | up |
| CHST2 | 1.952319376 | 9.27E-18 | 1.04E-15 | up |
| GUCY1B3 | 1.951798686 | 6.09E-18 | 7.22E-16 | up |
| TAGAP | 1.947466174 | 7.73E-12 | 1.65E-10 | up |
| SNX10 | 1.945841872 | 4.26E-16 | 2.96E-14 | up |
| CCL19 | 1.943751724 | 6.38E-09 | 6.63E-08 | up |
| FCRL5 | 1.941379639 | 2.71E-11 | 5.03E-10 | up |
| HLA-DOB | 1.934481609 | 3.97E-12 | 9.27E-11 | up |
| HAPLN3 | 1.929074384 | 2.10E-17 | 2.10E-15 | up |
| FAIM2 | 1.927684072 | 5.53E-19 | 8.53E-17 | up |
| IRAK3 | 1.924413629 | 3.23E-25 | 2.93E-22 | up |
| IGHV1-69 | 1.921398522 | 1.05E-07 | 8.21E-07 | up |
| MIR155HG | 1.91675665 | 6.52E-11 | 1.10E-09 | up |
| IGDCC4 | 1.916469951 | 1.88E-20 | 4.52E-18 | up |
| NCF2 | 1.91433711 | 2.47E-14 | 1.01E-12 | up |
| LAX1 | 1.911008539 | 5.78E-14 | 2.15E-12 | up |
| COL12A1 | 1.909348276 | 2.33E-10 | 3.38E-09 | up |
| TMEM163 | 1.892721182 | 2.39E-10 | 3.45E-09 | up |
| KIAA0802 | 1.881420361 | 5.67E-17 | 4.95E-15 | up |
| HGF | 1.874218227 | 1.89E-11 | 3.61E-10 | up |
| BGN | 1.873513136 | 1.12E-14 | 5.12E-13 | up |
| PLAU | 1.861776683 | 1.64E-16 | 1.23E-14 | up |
| CTSE | 1.856209852 | 4.39E-13 | 1.32E-11 | up |
| PLA1A | 1.855809031 | 1.73E-15 | 9.86E-14 | up |
| CTHRC1 | 1.851310673 | 3.45E-11 | 6.24E-10 | up |
| ANXA1 | 1.848134975 | 1.32E-19 | 2.39E-17 | up |
| IL33 | 1.842593103 | 9.49E-13 | 2.59E-11 | up |
| ITGAX | 1.84113514 | 4.91E-14 | 1.86E-12 | up |
| FCRL3 | 1.835416749 | 3.86E-08 | 3.36E-07 | up |
| PHLDA1 | 1.833250082 | 1.07E-15 | 6.43E-14 | up |
| ARNTL2 | 1.825833498 | 5.90E-26 | 7.35E-23 | up |
| ADAMTS1 | 1.823177668 | 5.70E-17 | 4.95E-15 | up |
| KIAA0125 | 1.80634647 | 9.08E-10 | 1.15E-08 | up |
| EGR3 | 1.805524138 | 1.30E-10 | 2.02E-09 | up |
| PTGES | 1.795101149 | 6.68E-12 | 1.45E-10 | up |
| P2RX5 | 1.794002299 | 8.80E-10 | 1.12E-08 | up |
| STEAP4 | 1.778513465 | 9.56E-13 | 2.61E-11 | up |
| GPR176 | 1.776346962 | 8.87E-15 | 4.15E-13 | up |
| SLC16A4 | 1.775133662 | 5.23E-10 | 7.01E-09 | up |
| CATSPERB | 1.773389163 | 3.50E-12 | 8.29E-11 | up |
| AIM2 | 1.762101642 | 1.24E-13 | 4.28E-12 | up |
| SDR16C5 | 1.7571133 | 1.79E-16 | 1.33E-14 | up |
| CD79A | 1.754462233 | 1.02E-12 | 2.77E-11 | up |
| PARP8 | 1.753121018 | 6.08E-28 | 1.21E-24 | up |
| CCL11 | 1.751577011 | 2.13E-15 | 1.17E-13 | up |
| ABCA12 | 1.750648112 | 6.43E-16 | 4.18E-14 | up |
| LAIR2 | 1.75049376 | 8.22E-09 | 8.35E-08 | up |
| CTSK | 1.749536617 | 5.85E-20 | 1.17E-17 | up |
| TREM1 | 1.746225287 | 4.37E-08 | 3.76E-07 | up |
| TRIB2 | 1.744483744 | 3.83E-17 | 3.62E-15 | up |
| S100P | 1.743041544 | 1.93E-25 | 1.92E-22 | up |
| G0S2 | 1.741816092 | 2.08E-11 | 3.94E-10 | up |
| ICAM1 | 1.739963711 | 2.75E-16 | 1.99E-14 | up |
| C4BPA | 1.737463547 | 3.90E-10 | 5.39E-09 | up |
| GEM | 1.737299343 | 3.77E-12 | 8.91E-11 | up |
| GBP1 | 1.735813629 | 2.09E-13 | 6.85E-12 | up |
| CHN1 | 1.734189327 | 2.45E-13 | 7.90E-12 | up |
| IGFBP5 | 1.733759934 | 1.63E-16 | 1.23E-14 | up |
| SAA4 | 1.731551724 | 8.49E-09 | 8.58E-08 | up |
| ECSCR | 1.731077997 | 3.20E-18 | 3.95E-16 | up |
| SLC7A11 | 1.728557964 | 5.93E-15 | 2.89E-13 | up |
| CR2 | 1.727654187 | 1.28E-05 | 6.35E-05 | up |
| CR1 | 1.721624302 | 1.46E-11 | 2.87E-10 | up |
| IFIT3 | 1.719785386 | 4.60E-13 | 1.38E-11 | up |
| INSL5 | 1.717290805 | 5.43E-05 | 0.000233392 | up |
| LRP8 | 1.708972906 | 2.17E-20 | 5.08E-18 | up |
| FAM129C | 1.707134483 | 2.11E-05 | 9.99E-05 | up |
| APOL1 | 1.705085878 | 9.77E-20 | 1.85E-17 | up |
| MYEOV | 1.704541379 | 8.53E-17 | 7.11E-15 | up |
| KCNJ15 | 1.70400312 | 3.79E-08 | 3.30E-07 | up |
| IGHD | 1.702421018 | 8.69E-06 | 4.48E-05 | up |
| HSD11B1 | 1.698267816 | 3.97E-09 | 4.30E-08 | up |
| ITGA5 | 1.696098686 | 1.28E-18 | 1.70E-16 | up |
| PLEK | 1.69585468 | 1.84E-11 | 3.52E-10 | up |
| GPR183 | 1.692981117 | 1.20E-14 | 5.40E-13 | up |
| SOD2 | 1.682309524 | 3.49E-12 | 8.29E-11 | up |
| CCL22 | 1.679062233 | 3.75E-11 | 6.70E-10 | up |
| CHRDL2 | 1.675349589 | 9.80E-11 | 1.57E-09 | up |
| THBD | 1.672966502 | 3.19E-14 | 1.25E-12 | up |
| VSIG1 | 1.672826273 | 3.32E-08 | 2.92E-07 | up |
| SLC7A5 | 1.672579146 | 1.41E-15 | 8.23E-14 | up |
| CDH11 | 1.667497865 | 8.72E-16 | 5.46E-14 | up |
| CCL4 | 1.662445977 | 6.76E-11 | 1.13E-09 | up |
| FCRL1 | 1.66235468 | 1.21E-06 | 7.48E-06 | up |
| CPXM1 | 1.661270936 | 1.29E-14 | 5.72E-13 | up |
| DOK3 | 1.660214286 | 1.63E-11 | 3.17E-10 | up |
| THY1 | 1.655615107 | 4.97E-17 | 4.49E-15 | up |
| GBP5 | 1.655571921 | 6.45E-13 | 1.84E-11 | up |
| GPR116 | 1.652065025 | 6.61E-13 | 1.88E-11 | up |
| DUSP4 | 1.650520361 | 2.55E-16 | 1.87E-14 | up |
| CLU | 1.647756322 | 1.61E-11 | 3.15E-10 | up |
| CCR6 | 1.645831199 | 2.76E-11 | 5.10E-10 | up |
| PCSK1 | 1.645668309 | 5.52E-09 | 5.82E-08 | up |
| OAS2 | 1.642208867 | 7.67E-14 | 2.76E-12 | up |
| LILRB2 | 1.638312972 | 5.87E-14 | 2.18E-12 | up |
| CD72 | 1.637640887 | 2.38E-09 | 2.73E-08 | up |
| BANK1 | 1.634540558 | 4.96E-08 | 4.21E-07 | up |
| MASP1 | 1.629737603 | 2.51E-08 | 2.28E-07 | up |
| MGC29506 | 1.629735304 | 1.00E-11 | 2.08E-10 | up |
| FAM65C | 1.628832841 | 2.44E-15 | 1.30E-13 | up |
| EOMES | 1.627153695 | 2.13E-15 | 1.17E-13 | up |
| DERL3 | 1.623843678 | 5.41E-12 | 1.21E-10 | up |
| SMARCB1 | 1.618533169 | 2.50E-12 | 6.18E-11 | up |
| TRIM22 | 1.615410837 | 6.85E-16 | 4.41E-14 | up |
| SPRR1B | 1.614813465 | 0.000156484 | 0.000596189 | up |
| RHOH | 1.610422167 | 6.06E-10 | 8.04E-09 | up |
| ELK3 | 1.608667816 | 3.02E-20 | 6.83E-18 | up |
| SOCS1 | 1.606898522 | 1.79E-16 | 1.33E-14 | up |
| LOXL2 | 1.60643908 | 1.03E-17 | 1.12E-15 | up |
| LRRK2 | 1.605751232 | 1.89E-08 | 1.77E-07 | up |
| CFP | 1.602587685 | 9.58E-15 | 4.46E-13 | up |
| COL6A3 | 1.600906404 | 4.18E-23 | 1.77E-20 | up |
| PTPRC | 1.597700821 | 8.36E-16 | 5.26E-14 | up |
| FILIP1L | 1.597150082 | 6.93E-20 | 1.35E-17 | up |
| GPX8 | 1.594785057 | 2.30E-15 | 1.25E-13 | up |
| CD19 | 1.590627915 | 6.27E-08 | 5.18E-07 | up |
| F2RL2 | 1.588534319 | 2.44E-15 | 1.30E-13 | up |
| CIITA | 1.586905583 | 1.51E-12 | 3.91E-11 | up |
| PTGDS | 1.585202299 | 8.46E-11 | 1.37E-09 | up |
| LOC399959 | 1.5838133 | 2.72E-13 | 8.69E-12 | up |
| PRR16 | 1.582890148 | 7.57E-12 | 1.63E-10 | up |
| VNN2 | 1.581033662 | 7.91E-09 | 8.05E-08 | up |
| ANGPTL2 | 1.576240722 | 1.03E-14 | 4.75E-13 | up |
| RSPO3 | 1.574349425 | 4.67E-11 | 8.15E-10 | up |
| LPCAT1 | 1.574271757 | 8.32E-28 | 1.45E-24 | up |
| IL7R | 1.574006568 | 1.40E-12 | 3.66E-11 | up |
| SP140 | 1.57220821 | 7.49E-13 | 2.09E-11 | up |
| COL4A1 | 1.570602627 | 3.14E-19 | 5.25E-17 | up |
| CD83 | 1.566859278 | 8.48E-10 | 1.08E-08 | up |
| KLHL5 | 1.560417241 | 5.67E-17 | 4.95E-15 | up |
| FCGR1B | 1.560240722 | 7.04E-11 | 1.17E-09 | up |
| TCL1A | 1.559442857 | 9.57E-05 | 0.000385067 | up |
| PLA2G2A | 1.557522332 | 1.75E-18 | 2.31E-16 | up |
| C2 | 1.55410509 | 5.17E-24 | 2.94E-21 | up |
| NOS2 | 1.552404105 | 1.30E-10 | 2.02E-09 | up |
| GUCY1A3 | 1.5503 | 3.93E-11 | 6.98E-10 | up |
| ROBO1 | 1.548346962 | 1.46E-14 | 6.44E-13 | up |
| GAS1 | 1.547815435 | 7.03E-05 | 0.000293751 | up |
| SLAMF6 | 1.547371757 | 2.30E-12 | 5.72E-11 | up |
| ST3GAL1 | 1.547027422 | 1.64E-15 | 9.42E-14 | up |
| MIAT | 1.545470608 | 2.77E-10 | 3.96E-09 | up |
| BHLHE22 | 1.543334319 | 4.93E-11 | 8.55E-10 | up |
| SLA | 1.542040558 | 6.05E-17 | 5.22E-15 | up |
| MME | 1.541625452 | 8.97E-10 | 1.14E-08 | up |
| IKBIP | 1.539170936 | 1.09E-18 | 1.49E-16 | up |
| CSF2RB | 1.536724466 | 5.40E-10 | 7.22E-09 | up |
| LILRA2 | 1.536102627 | 6.40E-10 | 8.44E-09 | up |
| CLEC5A | 1.532699672 | 2.46E-08 | 2.24E-07 | up |
| SLCO1B3 | 1.529202627 | 1.95E-07 | 1.43E-06 | up |
| SELE | 1.528252874 | 5.41E-06 | 2.93E-05 | up |
| EBF1 | 1.517796223 | 1.65E-09 | 1.97E-08 | up |
| EIF5A2 | 1.517627258 | 3.81E-18 | 4.55E-16 | up |
| DYSF | 1.51680624 | 1.91E-14 | 8.06E-13 | up |
| AREG | 1.514506404 | 3.16E-12 | 7.64E-11 | up |
| ELTD1 | 1.511999015 | 2.08E-14 | 8.72E-13 | up |
| MGP | 1.511052545 | 3.85E-13 | 1.18E-11 | up |
| VWF | 1.506830049 | 1.34E-11 | 2.68E-10 | up |
| ACSL4 | 1.503851888 | 5.76E-12 | 1.28E-10 | up |
| SAMD9L | 1.502270936 | 6.32E-18 | 7.40E-16 | up |
| BEST4 | -1.509373727 | 9.83E-08 | 7.77E-07 | down |
| AIFM3 | -1.509957635 | 4.20E-11 | 7.39E-10 | down |
| C10orf116 | -1.512471921 | 9.99E-21 | 2.52E-18 | down |
| EYA2 | -1.513284236 | 4.58E-13 | 1.37E-11 | down |
| PXMP2 | -1.517189163 | 3.43E-14 | 1.34E-12 | down |
| GDPD2 | -1.524343186 | 8.42E-13 | 2.32E-11 | down |
| CDHR1 | -1.532785878 | 6.67E-07 | 4.36E-06 | down |
| EFNA1 | -1.536175862 | 5.08E-13 | 1.49E-11 | down |
| C10orf108 | -1.538797537 | 4.06E-15 | 2.06E-13 | down |
| FAM55D | -1.544185878 | 2.44E-07 | 1.74E-06 | down |
| SLC39A2 | -1.545965025 | 3.31E-19 | 5.44E-17 | down |
| PLEKHG6 | -1.553013136 | 5.02E-13 | 1.48E-11 | down |
| ESPN | -1.555070279 | 1.88E-12 | 4.79E-11 | down |
| TMEM171 | -1.557557964 | 7.94E-14 | 2.85E-12 | down |
| NEURL1B | -1.558563218 | 1.16E-19 | 2.14E-17 | down |
| GSTA1 | -1.559916092 | 8.32E-08 | 6.68E-07 | down |
| LOC100287411 | -1.568869951 | 3.64E-15 | 1.87E-13 | down |
| LOC727916 | -1.573654023 | 2.95E-13 | 9.30E-12 | down |
| ANK3 | -1.580686864 | 8.68E-19 | 1.25E-16 | down |
| LIPC | -1.584017898 | 1.59E-22 | 5.85E-20 | down |
| GCNT2 | -1.592902135 | 7.94E-21 | 2.03E-18 | down |
| FMO4 | -1.599639901 | 3.98E-17 | 3.72E-15 | down |
| ETNK1 | -1.605910181 | 3.24E-21 | 8.84E-19 | down |
| LOC100286922 | -1.608550575 | 3.55E-17 | 3.41E-15 | down |
| PPARG | -1.614452709 | 3.36E-13 | 1.04E-11 | down |
| PIGZ | -1.620480131 | 5.06E-10 | 6.81E-09 | down |
| SGK2 | -1.623861084 | 1.89E-12 | 4.80E-11 | down |
| HEPACAM2 | -1.624314778 | 1.33E-05 | 6.58E-05 | down |
| VSTM2A | -1.625163711 | 1.89E-15 | 1.07E-13 | down |
| EXPH5 | -1.628878325 | 2.79E-10 | 3.98E-09 | down |
| EDN3 | -1.629215764 | 1.27E-09 | 1.54E-08 | down |
| C1orf175 | -1.63278358 | 2.27E-13 | 7.38E-12 | down |
| MEP1A | -1.644277668 | 1.35E-10 | 2.09E-09 | down |
| RHOU | -1.648068309 | 1.82E-18 | 2.38E-16 | down |
| LRRC19 | -1.659251232 | 2.09E-10 | 3.07E-09 | down |
| LDHD | -1.66052266 | 1.00E-15 | 6.11E-14 | down |
| USP2 | -1.664816749 | 2.12E-10 | 3.10E-09 | down |
| KIAA1984 | -1.677204598 | 4.02E-14 | 1.54E-12 | down |
| INPP5J | -1.683767816 | 1.02E-18 | 1.43E-16 | down |
| TMEM56 | -1.684913957 | 4.47E-16 | 3.07E-14 | down |
| CCNJL | -1.685094089 | 4.69E-19 | 7.53E-17 | down |
| FAM82A1 | -1.686720197 | 4.79E-15 | 2.39E-13 | down |
| MB | -1.687355993 | 6.37E-09 | 6.62E-08 | down |
| TMEM63C | -1.690029392 | 3.64E-17 | 3.47E-15 | down |
| PTPRR | -1.690349097 | 8.84E-09 | 8.90E-08 | down |
| METTL7B | -1.699011987 | 3.46E-12 | 8.21E-11 | down |
| ISX | -1.702941051 | 1.04E-08 | 1.04E-07 | down |
| FLJ22763 | -1.703845649 | 2.02E-11 | 3.84E-10 | down |
| ZNF575 | -1.704782923 | 1.05E-21 | 3.11E-19 | down |
| ENPP1 | -1.706773399 | 2.57E-19 | 4.34E-17 | down |
| LOC100128893 | -1.714000821 | 1.21E-12 | 3.21E-11 | down |
| NR1H4 | -1.71406601 | 2.87E-11 | 5.28E-10 | down |
| SCUBE2 | -1.7148133 | 4.74E-24 | 2.78E-21 | down |
| LOC100422737 | -1.716173071 | 6.28E-15 | 3.04E-13 | down |
| CDKN2B | -1.718584729 | 3.00E-11 | 5.49E-10 | down |
| PADI2 | -1.723572578 | 3.97E-14 | 1.52E-12 | down |
| LOC646627 | -1.724451067 | 3.76E-08 | 3.28E-07 | down |
| C7orf10 | -1.725023317 | 1.62E-17 | 1.69E-15 | down |
| PBLD | -1.7269711 | 1.85E-14 | 7.85E-13 | down |
| MS4A12 | -1.734644663 | 4.19E-07 | 2.85E-06 | down |
| UGT2B15 | -1.739174384 | 1.11E-06 | 6.90E-06 | down |
| RPS6KA6 | -1.742337438 | 1.05E-17 | 1.14E-15 | down |
| FLJ35024 | -1.745970936 | 1.24E-18 | 1.67E-16 | down |
| YBX2 | -1.74792069 | 1.29E-10 | 2.01E-09 | down |
| VLDLR | -1.749346798 | 4.63E-24 | 2.78E-21 | down |
| FRMD1 | -1.758215271 | 1.73E-17 | 1.76E-15 | down |
| HSD17B2 | -1.760987192 | 2.90E-15 | 1.51E-13 | down |
| VIPR1 | -1.767199836 | 6.50E-11 | 1.09E-09 | down |
| MS4A8B | -1.768694581 | 1.05E-10 | 1.68E-09 | down |
| TMEM37 | -1.770342693 | 6.69E-12 | 1.45E-10 | down |
| PAQR5 | -1.770470936 | 5.49E-20 | 1.12E-17 | down |
| SLC25A34 | -1.776747783 | 3.53E-20 | 7.89E-18 | down |
| SLC39A5 | -1.786559442 | 4.60E-14 | 1.76E-12 | down |
| TRHDE | -1.787890969 | 1.83E-25 | 1.92E-22 | down |
| CNGA1 | -1.793061412 | 3.34E-21 | 8.97E-19 | down |
| PHLPP2 | -1.799260427 | 3.42E-18 | 4.18E-16 | down |
| B4GALNT2 | -1.818643678 | 6.10E-24 | 3.38E-21 | down |
| SLC4A4 | -1.822183087 | 1.30E-08 | 1.26E-07 | down |
| LOC25845 | -1.825434811 | 2.91E-15 | 1.51E-13 | down |
| SLC16A9 | -1.826910673 | 6.40E-12 | 1.40E-10 | down |
| PPARGC1A | -1.833709195 | 6.16E-13 | 1.77E-11 | down |
| WDR78 | -1.847634319 | 1.38E-19 | 2.48E-17 | down |
| TMEM20 | -1.849355829 | 1.28E-21 | 3.69E-19 | down |
| NPY | -1.893928243 | 1.01E-17 | 1.11E-15 | down |
| DPP10 | -1.896852545 | 1.09E-32 | 5.24E-29 | down |
| LOC100288092 | -1.897361412 | 1.09E-14 | 4.98E-13 | down |
| OSTBETA | -1.927264532 | 5.36E-13 | 1.57E-11 | down |
| TUBAL3 | -1.946329885 | 2.06E-14 | 8.68E-13 | down |
| SLC23A3 | -1.949557471 | 1.61E-23 | 7.81E-21 | down |
| ANKRD43 | -1.967200493 | 1.25E-11 | 2.52E-10 | down |
| CYP4F2 | -1.980938916 | 4.77E-16 | 3.26E-14 | down |
| UGT1A6 | -1.986738424 | 9.66E-16 | 5.92E-14 | down |
| GUCA2A | -1.999787685 | 6.22E-10 | 8.22E-09 | down |
| ACSF2 | -2.013490805 | 5.97E-23 | 2.43E-20 | down |
| PRAP1 | -2.019073071 | 5.52E-11 | 9.49E-10 | down |
| ANPEP | -2.019960591 | 8.06E-11 | 1.31E-09 | down |
| FAM189A1 | -2.052004269 | 9.03E-18 | 1.02E-15 | down |
| PKIB | -2.060541215 | 7.21E-12 | 1.55E-10 | down |
| DHRS11 | -2.065951724 | 1.60E-16 | 1.21E-14 | down |
| ABCB1 | -2.0670867 | 1.71E-17 | 1.76E-15 | down |
| PDE6A | -2.070992611 | 7.89E-25 | 6.04E-22 | down |
| CAPN13 | -2.073469458 | 1.35E-10 | 2.09E-09 | down |
| PCDH20 | -2.09722775 | 1.70E-09 | 2.02E-08 | down |
| HSPB3 | -2.111409195 | 4.49E-24 | 2.78E-21 | down |
| FAM5C | -2.128840394 | 3.30E-18 | 4.06E-16 | down |
| TEX11 | -2.141362562 | 1.60E-17 | 1.67E-15 | down |
| RUNDC3B | -2.16476486 | 6.71E-24 | 3.49E-21 | down |
| CDKN2BAS | -2.168202463 | 2.70E-22 | 9.11E-20 | down |
| CA1 | -2.177052053 | 1.53E-06 | 9.31E-06 | down |
| WSCD1 | -2.230492118 | 6.83E-24 | 3.49E-21 | down |
| ADH1C | -2.236926765 | 9.44E-10 | 1.19E-08 | down |
| LAMA1 | -2.284044499 | 2.56E-13 | 8.23E-12 | down |
| APOBEC3B | -2.352381445 | 1.48E-24 | 1.02E-21 | down |
| MT1M | -2.363885386 | 6.86E-11 | 1.14E-09 | down |
| UGT2B17 | -2.373620197 | 2.28E-05 | 0.00010692 | down |
| SLC23A1 | -2.397701314 | 1.32E-32 | 5.24E-29 | down |
| SLC3A1 | -2.409644663 | 1.08E-14 | 4.96E-13 | down |
| BEST2 | -2.431984565 | 8.03E-13 | 2.23E-11 | down |
| AQP7 | -2.452370772 | 2.03E-29 | 4.50E-26 | down |
| SLC26A2 | -2.453400164 | 7.16E-16 | 4.57E-14 | down |
| DEFB1 | -2.457067816 | 1.74E-17 | 1.77E-15 | down |
| SLC17A4 | -2.493070115 | 1.24E-15 | 7.39E-14 | down |
| TRPM6 | -2.533384072 | 2.07E-12 | 5.23E-11 | down |
| CHP2 | -2.580467652 | 2.24E-13 | 7.29E-12 | down |
| SLC30A10 | -2.613824138 | 6.05E-13 | 1.75E-11 | down |
| NPY1R | -2.626588013 | 1.24E-24 | 9.11E-22 | down |
| FLJ32063 | -2.686870443 | 3.44E-14 | 1.34E-12 | down |
| PITX2 | -2.777535304 | 1.95E-20 | 4.62E-18 | down |
| HSD3B2 | -2.789092282 | 6.66E-19 | 9.90E-17 | down |
| CWH43 | -2.807515599 | 9.45E-12 | 1.97E-10 | down |
| GUCA2B | -2.820729885 | 4.07E-12 | 9.48E-11 | down |
| CYP2B7P1 | -2.843197701 | 4.56E-23 | 1.89E-20 | down |
| UGT2A3 | -2.890092939 | 1.98E-18 | 2.56E-16 | down |
| PNLIPRP2 | -2.992193268 | 5.58E-14 | 2.09E-12 | down |
| CYP2B6 | -3.014818719 | 3.67E-20 | 8.04E-18 | down |
| MEP1B | -3.015463218 | 4.99E-25 | 4.32E-22 | down |
| SLC38A4 | -3.045252381 | 1.70E-30 | 4.23E-27 | down |
| GBA3 | -3.078827422 | 1.78E-22 | 6.34E-20 | down |
| ABCG2 | -3.196533333 | 6.51E-20 | 1.28E-17 | down |
| HMGCS2 | -3.398166995 | 1.28E-14 | 5.71E-13 | down |
| LOC389023 | -3.51543711 | 6.76E-39 | 6.73E-35 | down |
| PCK1 | -3.631396223 | 2.69E-13 | 8.60E-12 | down |
| CLDN8 | -3.683299836 | 5.08E-10 | 6.83E-09 | down |
| OSTalpha | -3.745492775 | 3.45E-22 | 1.09E-19 | down |
| AQP8 | -4.773067488 | 9.51E-22 | 2.91E-19 | down |

Supplementary Table S3: Hub targets related to active ingredients

| Molecule ID | Molecule Name | Hub Target | Source |
| --- | --- | --- | --- |
| MOL000098 | quercetin | CXCL8, CCL2, ICAM1, IL-1β, MMP9, PTGS2, MMP1, SELE, CXCL10, MMP3 | HuangBai,HuangLian,ZhiZi |
| MOL000173 | wogonin | CXCL8, MMP9, PTGS2, CCL2, MMP1 | HuangQin |
| MOL000422 | kaempferol | ICAM1, PTGS2, SELE | ZhiZi |
| MOL000525 | Norwogonin | PTGS2 | HuangQin |
| MOL002933 | 5,7,4'-Trihydroxy-8-methoxyflavone | PTGS2 | HuangQin |
| MOL002934 | NEOBAICALEIN | PTGS2 | HuangQin |
| MOL003095 | 5-hydroxy-7-methoxy-2-(3,4,5-trimethoxyphenyl) chromone | PTGS2 | ZhiZi |
| MOL008206 | Moslosooflavone | PTGS2 | HuangQin |
| MOL000449 | Stigmasterol | PTGS2 | HuangBai,HuangQin,ZhiZi |
| MOL002662 | rutaecarpine | PTGS2 | HuangBai |
| MOL000552 | 5,2'-Dihydroxy-6,7,8-trimethoxyflavone | PTGS2 | HuangQin |
| MOL000785 | palmatine | PTGS2 | HuangBai,HuangLian |
| MOL001454 | berberine | PTGS2 | HuangBai,HuangLian |
| MOL001458 | coptisine | PTGS2 | HuangBai,HuangLian,HuangQin |
| MOL001689 | acacetin | PTGS2 | HuangQin |
| MOL002668 | Worenine | PTGS2 | HuangBai,HuangLian |
| MOL002894 | berberrubine | PTGS2 | HuangBai,HuangLian |
| MOL002897 | epiberberine | PTGS2 | HuangLian,HuangQin |
| MOL002904 | Berlambine | PTGS2 | HuangLian |
| MOL002909 | 5,7,2,5-tetrahydroxy-8,6-dimethoxyflavone | PTGS2 | HuangQin |
| MOL002915 | Salvigenin | PTGS2 | HuangQin |
| MOL002917 | 5,2',6'-Trihydroxy-7,8-dimethoxyflavone | PTGS2 | HuangQin |
| MOL002927 | Skullcapflavone II | PTGS2 | HuangQin |
| MOL002928 | oroxylin a | PTGS2 | HuangQin |
| MOL002932 | Panicolin | PTGS2 | HuangQin |
| MOL006422 | thalifendine | PTGS2 | HuangBai |
| MOL007245 | 3-Methylkempferol | PTGS2 | ZhiZi |
| MOL012266 | rivularin | PTGS2 | HuangQin |
| MOL002651 | Dehydrotanshinone II A | PTGS2 | HuangBai |
| MOL000073 | ent-Epicatechin | PTGS2 | HuangQin |
| MOL000228 | (2R)-7-hydroxy-5-methoxy-2-phenylchroman-4-one | PTGS2 | HuangQin |
| MOL000358 | beta-sitosterol | PTGS2 | HuangBai,HuangQin,ZhiZi |
| MOL000787 | Fumarine | PTGS2 | HuangBai |
| MOL000790 | Isocorypalmine | PTGS2 | HuangBai |
| MOL001131 | phellamurin_qt | PTGS2 | HuangBai |
| MOL001406 | crocetin | PTGS2 | ZhiZi |
| MOL001455 | (S)-Canadine | PTGS2 | HuangBai |
| MOL001494 | Mandenol | PTGS2 | ZhiZi |
| MOL001941 | Ammidin | PTGS2 | ZhiZi |
| MOL001942 | isoimperatorin | PTGS2 | ZhiZi |
| MOL002641 | Phellavin_qt | PTGS2 | HuangBai |
| MOL002644 | Phellopterin | PTGS2 | HuangBai |
| MOL002666 | Chelerythrine | PTGS2 | HuangBai |
| MOL002670 | Cavidine | PTGS2 | HuangBai |
| MOL002714 | baicalein | PTGS2 | HuangQin |
| MOL002903 | (R)-Canadine | PTGS2 | HuangLian |
| MOL002910 | Carthamidin | PTGS2 | HuangQin |
| MOL002913 | Dihydrobaicalin_qt | PTGS2 | HuangQin |
| MOL002914 | Eriodyctiol (flavanone) | PTGS2 | HuangQin |
| MOL002925 | 5,7,2',6'-Tetrahydroxyflavone | PTGS2 | HuangQin |
| MOL002937 | DIHYDROOROXYLIN | PTGS2 | HuangQin |
| MOL004561 | Sudan III | PTGS2 | ZhiZi |
| MOL012245 | 5,7,4'-trihydroxy-6-methoxyflavanone | PTGS2 | HuangQin |
| MOL012246 | 5,7,4'-trihydroxy-8-methoxyflavanone | PTGS2 | HuangQin |

Supplementary Table S4: The GO-BP terms enriched by target genes.

| ID | Description | GeneRatio | p.adjust | Count |
| --- | --- | --- | --- | --- |
| GO:0032496 | response to lipopolysaccharide | 7/10 | 4.89E-08 | 7 |
| GO:0002237 | response to molecule of bacterial origin | 7/10 | 4.89E-08 | 7 |
| GO:0150077 | regulation of neuroinflammatory response | 4/10 | 1.73E-06 | 4 |
| GO:0070555 | response to interleukin-1 | 5/10 | 7.04E-06 | 5 |
| GO:0002685 | regulation of leukocyte migration | 5/10 | 7.04E-06 | 5 |
| GO:0150076 | neuroinflammatory response | 4/10 | 7.04E-06 | 4 |
| GO:0071216 | cellular response to biotic stimulus | 5/10 | 1.02E-05 | 5 |
| GO:0030593 | neutrophil chemotaxis | 4/10 | 1.83E-05 | 4 |
| GO:0034612 | response to tumor necrosis factor | 5/10 | 3.01E-05 | 5 |
| GO:1990266 | neutrophil migration | 4/10 | 3.01E-05 | 4 |
| GO:0045429 | positive regulation of nitric oxide biosynthetic process | 3/10 | 9.16E-05 | 3 |
| GO:1904407 | positive regulation of nitric oxide metabolic process | 3/10 | 9.16E-05 | 3 |
| GO:0030574 | collagen catabolic process | 3/10 | 9.16E-05 | 3 |
| GO:0071347 | cellular response to interleukin-1 | 4/10 | 9.16E-05 | 4 |
| GO:2000377 | regulation of reactive oxygen species metabolic process | 4/10 | 0.000124295 | 4 |
| GO:1904645 | response to amyloid-beta | 3/10 | 0.000141931 | 3 |
| GO:0007566 | embryo implantation | 3/10 | 0.000151035 | 3 |
| GO:2001234 | negative regulation of apoptotic signaling pathway | 4/10 | 0.000176902 | 4 |
| GO:1903039 | positive regulation of leukocyte cell-cell adhesion | 4/10 | 0.000176902 | 4 |
| GO:0051924 | regulation of calcium ion transport | 4/10 | 0.000207368 | 4 |
| GO:0022617 | extracellular matrix disassembly | 3/10 | 0.000282963 | 3 |
| GO:0072593 | reactive oxygen species metabolic process | 4/10 | 0.000299321 | 4 |
| GO:2001057 | reactive nitrogen species metabolic process | 3/10 | 0.000306283 | 3 |
| GO:0001660 | fever generation | 2/10 | 0.000306283 | 2 |
| GO:0070098 | chemokine-mediated signaling pathway | 3/10 | 0.000306283 | 3 |
| GO:0031652 | positive regulation of heat generation | 2/10 | 0.000357 | 2 |
| GO:1990868 | response to chemokine | 3/10 | 0.000373169 | 3 |
| GO:0031392 | regulation of prostaglandin biosynthetic process | 2/10 | 0.000394245 | 2 |
| GO:0002526 | acute inflammatory response | 3/10 | 0.000473188 | 3 |
| GO:0032963 | collagen metabolic process | 3/10 | 0.000489775 | 3 |
| GO:0007159 | leukocyte cell-cell adhesion | 4/10 | 0.000515457 | 4 |
| GO:0006959 | humoral immune response | 4/10 | 0.000570364 | 4 |
| GO:0045765 | regulation of angiogenesis | 4/10 | 0.000658251 | 4 |
| GO:0003158 | endothelium development | 3/10 | 0.000690392 | 3 |
| GO:0060353 | regulation of cell adhesion molecule production | 2/10 | 0.000768863 | 2 |
| GO:0006816 | calcium ion transport | 4/10 | 0.000768863 | 4 |
| GO:0036499 | PERK-mediated unfolded protein response | 2/10 | 0.000793032 | 2 |
| GO:0140467 | integrated stress response signaling | 2/10 | 0.000793032 | 2 |
| GO:0060352 | cell adhesion molecule production | 2/10 | 0.000836734 | 2 |
| GO:0010575 | positive regulation of vascular endothelial growth factor production | 2/10 | 0.001233525 | 2 |
| GO:0051968 | positive regulation of synaptic transmission | 2/10 | 0.001538707 | 2 |
| GO:0033198 | response to ATP | 2/10 | 0.00166515 | 2 |
| GO:0045907 | positive regulation of vasoconstriction | 2/10 | 0.00166515 | 2 |
| GO:0009612 | response to mechanical stimulus | 3/10 | 0.001759527 | 3 |
| GO:0033280 | response to vitamin D | 2/10 | 0.001808611 | 2 |
| GO:0071241 | cellular response to inorganic substance | 3/10 | 0.001988537 | 3 |
| GO:0044706 | multi-multicellular organism process | 3/10 | 0.002102353 | 3 |
| GO:0050920 | regulation of chemotaxis | 3/10 | 0.002163435 | 3 |
| GO:0009743 | response to carbohydrate | 3/10 | 0.002253763 | 3 |
| GO:0061028 | establishment of endothelial barrier | 2/10 | 0.002728213 | 2 |
| GO:0007157 | heterophilic cell-cell adhesion via plasma membrane cell adhesion molecules | 2/10 | 0.002793347 | 2 |
| GO:0031334 | positive regulation of protein-containing complex assembly | 3/10 | 0.002863186 | 3 |
| GO:0006692 | prostanoid metabolic process | 2/10 | 0.002923575 | 2 |
| GO:0006693 | prostaglandin metabolic process | 2/10 | 0.002923575 | 2 |
| GO:0006984 | ER-nucleus signaling pathway | 2/10 | 0.003138568 | 2 |
| GO:0071356 | cellular response to tumor necrosis factor | 3/10 | 0.003957993 | 3 |
| GO:0070372 | regulation of ERK1 and ERK2 cascade | 3/10 | 0.004209396 | 3 |
| GO:2000351 | regulation of endothelial cell apoptotic process | 2/10 | 0.004211516 | 2 |
| GO:0010573 | vascular endothelial growth factor production | 2/10 | 0.004441883 | 2 |
| GO:0046824 | positive regulation of nucleocytoplasmic transport | 2/10 | 0.004542761 | 2 |
| GO:0050918 | positive chemotaxis | 2/10 | 0.004600863 | 2 |
| GO:0070371 | ERK1 and ERK2 cascade | 3/10 | 0.004600863 | 3 |
| GO:0071496 | cellular response to external stimulus | 3/10 | 0.004600863 | 3 |
| GO:0050766 | positive regulation of phagocytosis | 2/10 | 0.004600863 | 2 |
| GO:0072577 | endothelial cell apoptotic process | 2/10 | 0.004600863 | 2 |
| GO:0019058 | viral life cycle | 3/10 | 0.00516182 | 3 |
| GO:1904062 | regulation of cation transmembrane transport | 3/10 | 0.00538792 | 3 |
| GO:0061844 | antimicrobial humoral immune response mediated by antimicrobial peptide | 2/10 | 0.00538792 | 2 |
| GO:0062197 | cellular response to chemical stress | 3/10 | 0.005739753 | 3 |
| GO:0071260 | cellular response to mechanical stimulus | 2/10 | 0.00580456 | 2 |
| GO:0010038 | response to metal ion | 3/10 | 0.005936805 | 3 |
| GO:0043154 | negative regulation of cysteine-type endopeptidase activity involved in apoptotic process | 2/10 | 0.006619855 | 2 |
| GO:0046889 | positive regulation of lipid biosynthetic process | 2/10 | 0.00699374 | 2 |
| GO:0042493 | response to drug | 3/10 | 0.007308535 | 3 |
| GO:0033273 | response to vitamin | 2/10 | 0.007372802 | 2 |
| GO:0035249 | synaptic transmission | 2/10 | 0.007979779 | 2 |
| GO:0048661 | positive regulation of smooth muscle cell proliferation | 2/10 | 0.008821453 | 2 |
| GO:1901570 | fatty acid derivative biosynthetic process | 2/10 | 0.008821453 | 2 |
| GO:0002456 | T cell mediated immunity | 2/10 | 0.008934605 | 2 |
| GO:0019233 | sensory perception of pain | 2/10 | 0.009219276 | 2 |
| GO:0009314 | response to radiation | 3/10 | 0.00927231 | 3 |
| GO:0043434 | response to peptide hormone | 3/10 | 0.00927231 | 3 |
| GO:0006979 | response to oxidative stress | 3/10 | 0.009645084 | 3 |
| GO:0031623 | receptor internalization | 2/10 | 0.010187003 | 2 |
| GO:0042110 | T cell activation | 3/10 | 0.010782147 | 3 |
| GO:0034605 | cellular response to heat | 2/10 | 0.01133538 | 2 |
| GO:0046683 | response to organophosphorus | 2/10 | 0.012784874 | 2 |
| GO:0035296 | regulation of tube diameter | 2/10 | 0.014257612 | 2 |
| GO:0035150 | regulation of tube size | 2/10 | 0.014257612 | 2 |
| GO:0044344 | cellular response to fibroblast growth factor stimulus | 2/10 | 0.01446073 | 2 |
| GO:0044409 | entry into host | 2/10 | 0.01446073 | 2 |
| GO:0051384 | response to glucocorticoid | 2/10 | 0.01446073 | 2 |
| GO:0014074 | response to purine-containing compound | 2/10 | 0.014772914 | 2 |
| GO:0071774 | response to fibroblast growth factor | 2/10 | 0.015402259 | 2 |
| GO:0062013 | positive regulation of small molecule metabolic process | 2/10 | 0.015520911 | 2 |
| GO:0008360 | regulation of cell shape | 2/10 | 0.015838186 | 2 |
| GO:0051092 | positive regulation of NF-kappaB transcription factor activity | 2/10 | 0.016276007 | 2 |
| GO:0030856 | regulation of epithelial cell differentiation | 2/10 | 0.017871907 | 2 |
| GO:0048659 | smooth muscle cell proliferation | 2/10 | 0.018177302 | 2 |
| GO:1901568 | fatty acid derivative metabolic process | 2/10 | 0.018177302 | 2 |
| GO:0010634 | positive regulation of epithelial cell migration | 2/10 | 0.018295938 | 2 |
| GO:0071346 | cellular response to interferon-gamma | 2/10 | 0.019355921 | 2 |
| GO:0090316 | positive regulation of intracellular protein transport | 2/10 | 0.019355921 | 2 |
| GO:0006986 | response to unfolded protein | 2/10 | 0.019474928 | 2 |
| GO:0010565 | regulation of cellular ketone metabolic process | 2/10 | 0.020553582 | 2 |
| GO:0003018 | vascular process in circulatory system | 2/10 | 0.020672818 | 2 |
| GO:0043112 | receptor metabolic process | 2/10 | 0.02220432 | 2 |
| GO:0071456 | cellular response to hypoxia | 2/10 | 0.023690167 | 2 |
| GO:0002703 | regulation of leukocyte mediated immunity | 2/10 | 0.023690167 | 2 |
| GO:0002291 | T cell activation via T cell receptor contact with antigen bound to MHC molecule on antigen presenting cell | 1/10 | 0.023690167 | 1 |
| GO:0019062 | virion attachment to host cell | 1/10 | 0.023690167 | 1 |
| GO:0032308 | positive regulation of prostaglandin secretion | 1/10 | 0.023690167 | 1 |
| GO:0033083 | regulation of immature T cell proliferation | 1/10 | 0.023690167 | 1 |
| GO:0034350 | regulation of glial cell apoptotic process | 1/10 | 0.023690167 | 1 |
| GO:0050870 | positive regulation of T cell activation | 2/10 | 0.02410848 | 2 |
| GO:0033079 | immature T cell proliferation | 1/10 | 0.024272233 | 1 |
| GO:0042118 | endothelial cell activation | 1/10 | 0.025811777 | 1 |
| GO:0042368 | vitamin D biosynthetic process | 1/10 | 0.025811777 | 1 |
| GO:0000302 | response to reactive oxygen species | 2/10 | 0.026184512 | 2 |
| GO:0010623 | programmed cell death involved in cell development | 1/10 | 0.026829655 | 1 |
| GO:0016264 | gap junction assembly | 1/10 | 0.026829655 | 1 |
| GO:0033089 | positive regulation of T cell differentiation in thymus | 1/10 | 0.026829655 | 1 |
| GO:0033127 | regulation of histone phosphorylation | 1/10 | 0.026829655 | 1 |
| GO:0030213 | hyaluronan biosynthetic process | 1/10 | 0.027964674 | 1 |
| GO:0034356 | NAD biosynthesis via nicotinamide riboside salvage pathway | 1/10 | 0.027964674 | 1 |
| GO:0047484 | regulation of response to osmotic stress | 1/10 | 0.027964674 | 1 |
| GO:0051607 | defense response to virus | 2/10 | 0.028634957 | 2 |
| GO:0042180 | cellular ketone metabolic process | 2/10 | 0.028634957 | 2 |
| GO:0034349 | glial cell apoptotic process | 1/10 | 0.028634957 | 1 |
| GO:0042362 | fat-soluble vitamin biosynthetic process | 1/10 | 0.028634957 | 1 |
| GO:0051044 | positive regulation of membrane protein ectodomain proteolysis | 1/10 | 0.028634957 | 1 |
| GO:0060009 | Sertoli cell development | 1/10 | 0.028634957 | 1 |
| GO:0002523 | leukocyte migration involved in inflammatory response | 1/10 | 0.029438949 | 1 |
| GO:0051770 | positive regulation of nitric-oxide synthase biosynthetic process | 1/10 | 0.029438949 | 1 |
| GO:0071732 | cellular response to nitric oxide | 1/10 | 0.029438949 | 1 |
| GO:1900451 | positive regulation of glutamate receptor signaling pathway | 1/10 | 0.029438949 | 1 |
| GO:0015732 | prostaglandin transport | 1/10 | 0.029903302 | 1 |
| GO:0030730 | sequestering of triglyceride | 1/10 | 0.029903302 | 1 |
| GO:0045986 | negative regulation of smooth muscle contraction | 1/10 | 0.029903302 | 1 |
| GO:0090185 | negative regulation of kidney development | 1/10 | 0.029903302 | 1 |
| GO:0090336 | positive regulation of brown fat cell differentiation | 1/10 | 0.029903302 | 1 |
| GO:1903209 | positive regulation of oxidative stress-induced cell death | 1/10 | 0.029903302 | 1 |
| GO:0010832 | negative regulation of myotube differentiation | 1/10 | 0.030977147 | 1 |
| GO:0071318 | cellular response to ATP | 1/10 | 0.030977147 | 1 |
| GO:0071605 | monocyte chemotactic protein-1 production | 1/10 | 0.030977147 | 1 |
| GO:0032409 | regulation of transporter activity | 2/10 | 0.03144056 | 2 |
| GO:0002922 | positive regulation of humoral immune response | 1/10 | 0.03144056 | 1 |
| GO:0003159 | morphogenesis of an endothelium | 1/10 | 0.03144056 | 1 |
| GO:0045091 | regulation of single stranded viral RNA replication via double stranded DNA intermediate | 1/10 | 0.03144056 | 1 |
| GO:0051546 | keratinocyte migration | 1/10 | 0.03144056 | 1 |
| GO:0060252 | positive regulation of glial cell proliferation | 1/10 | 0.03144056 | 1 |
| GO:0061154 | endothelial tube morphogenesis | 1/10 | 0.03144056 | 1 |
| GO:1902170 | cellular response to reactive nitrogen species | 1/10 | 0.03144056 | 1 |
| GO:0034976 | response to endoplasmic reticulum stress | 2/10 | 0.03144056 | 2 |
| GO:0002726 | positive regulation of T cell cytokine production | 1/10 | 0.031868741 | 1 |
| GO:0002827 | positive regulation of T-helper 1 type immune response | 1/10 | 0.031868741 | 1 |
| GO:0051767 | nitric-oxide synthase biosynthetic process | 1/10 | 0.031868741 | 1 |
| GO:0071731 | response to nitric oxide | 1/10 | 0.031868741 | 1 |
| GO:0042063 | gliogenesis | 2/10 | 0.032420117 | 2 |
| GO:0030728 | ovulation | 1/10 | 0.032420117 | 1 |
| GO:0071498 | cellular response to fluid shear stress | 1/10 | 0.032420117 | 1 |
| GO:0042359 | vitamin D metabolic process | 1/10 | 0.033519605 | 1 |
| GO:1901739 | regulation of myoblast fusion | 1/10 | 0.033519605 | 1 |
| GO:1902884 | positive regulation of response to oxidative stress | 1/10 | 0.033519605 | 1 |
| GO:2000479 | regulation of cAMP-dependent protein kinase activity | 1/10 | 0.033519605 | 1 |
| GO:0007568 | aging | 2/10 | 0.033782898 | 2 |
| GO:0051235 | maintenance of location | 2/10 | 0.034382627 | 2 |
| GO:0035743 | CD4-positive | 1/10 | 0.034382627 | 1 |
| GO:0006898 | receptor-mediated endocytosis | 2/10 | 0.034966695 | 2 |
| GO:0002438 | acute inflammatory response to antigenic stimulus | 1/10 | 0.035224908 | 1 |
| GO:0050995 | negative regulation of lipid catabolic process | 1/10 | 0.035224908 | 1 |
| GO:0050863 | regulation of T cell activation | 2/10 | 0.035224908 | 2 |
| GO:1903829 | positive regulation of cellular protein localization | 2/10 | 0.036106257 | 2 |
| GO:0002092 | positive regulation of receptor internalization | 1/10 | 0.036106257 | 1 |
| GO:0045662 | negative regulation of myoblast differentiation | 1/10 | 0.036106257 | 1 |
| GO:0046697 | decidualization | 1/10 | 0.036106257 | 1 |
| GO:0009615 | response to virus | 2/10 | 0.037436377 | 2 |
| GO:0034698 | response to gonadotropin | 1/10 | 0.037436377 | 1 |
| GO:0048143 | astrocyte activation | 1/10 | 0.037436377 | 1 |
| GO:1900120 | regulation of receptor binding | 1/10 | 0.037436377 | 1 |
| GO:0006913 | nucleocytoplasmic transport | 2/10 | 0.037459437 | 2 |
| GO:0051222 | positive regulation of protein transport | 2/10 | 0.037459437 | 2 |
| GO:0051251 | positive regulation of lymphocyte activation | 2/10 | 0.037606536 | 2 |
| GO:0001666 | response to hypoxia | 2/10 | 0.037606536 | 2 |
| GO:0090200 | positive regulation of release of cytochrome c from mitochondria | 1/10 | 0.037606536 | 1 |
| GO:1902932 | positive regulation of alcohol biosynthetic process | 1/10 | 0.037606536 | 1 |
| GO:1905563 | negative regulation of vascular endothelial cell proliferation | 1/10 | 0.037606536 | 1 |
| GO:0002449 | lymphocyte mediated immunity | 2/10 | 0.038480059 | 2 |
| GO:0090132 | epithelium migration | 2/10 | 0.038526594 | 2 |
| GO:0002460 | adaptive immune response based on somatic recombination of immune receptors built from immunoglobulin superfamily domains | 2/10 | 0.038652823 | 2 |
| GO:1904951 | positive regulation of establishment of protein localization | 2/10 | 0.038652823 | 2 |
| GO:0035066 | positive regulation of histone acetylation | 1/10 | 0.038878363 | 1 |
| GO:1900027 | regulation of ruffle assembly | 1/10 | 0.038878363 | 1 |
| GO:0032770 | positive regulation of monooxygenase activity | 1/10 | 0.039436126 | 1 |
| GO:0045987 | positive regulation of smooth muscle contraction | 1/10 | 0.039436126 | 1 |
| GO:0071549 | cellular response to dexamethasone stimulus | 1/10 | 0.039436126 | 1 |
| GO:0006909 | phagocytosis | 2/10 | 0.039856669 | 2 |
| GO:1901889 | negative regulation of cell junction assembly | 1/10 | 0.040152272 | 1 |
| GO:0001975 | response to amphetamine | 1/10 | 0.041030008 | 1 |
| GO:0046627 | negative regulation of insulin receptor signaling pathway | 1/10 | 0.041030008 | 1 |
| GO:1901653 | cellular response to peptide | 2/10 | 0.041526658 | 2 |
| GO:0007202 | activation of phospholipase C activity | 1/10 | 0.041526658 | 1 |
| GO:0035633 | maintenance of blood-brain barrier | 1/10 | 0.041526658 | 1 |
| GO:0048566 | embryonic digestive tract development | 1/10 | 0.041526658 | 1 |
| GO:0071312 | cellular response to alkaloid | 1/10 | 0.042005722 | 1 |
| GO:0072525 | pyridine-containing compound biosynthetic process | 1/10 | 0.042005722 | 1 |
| GO:0043547 | positive regulation of GTPase activity | 2/10 | 0.042149461 | 2 |
| GO:0034405 | response to fluid shear stress | 1/10 | 0.042378871 | 1 |
| GO:1901099 | negative regulation of signal transduction in absence of ligand | 1/10 | 0.042378871 | 1 |
| GO:2000310 | regulation of NMDA receptor activity | 1/10 | 0.042378871 | 1 |
| GO:0030212 | hyaluronan metabolic process | 1/10 | 0.04327296 | 1 |
| GO:0090050 | positive regulation of cell migration involved in sprouting angiogenesis | 1/10 | 0.04327296 | 1 |
| GO:2000273 | positive regulation of signaling receptor activity | 1/10 | 0.04327296 | 1 |
| GO:0016572 | histone phosphorylation | 1/10 | 0.045556796 | 1 |
| GO:0032892 | positive regulation of organic acid transport | 1/10 | 0.045556796 | 1 |
| GO:0050691 | regulation of defense response to virus by host | 1/10 | 0.045556796 | 1 |
| GO:0051281 | positive regulation of release of sequestered calcium ion into cytosol | 1/10 | 0.045556796 | 1 |
| GO:0140353 | lipid export from cell | 1/10 | 0.045556796 | 1 |
| GO:0071548 | response to dexamethasone | 1/10 | 0.046263537 | 1 |
| GO:0072524 | pyridine-containing compound metabolic process | 1/10 | 0.046263537 | 1 |
| GO:0048608 | reproductive structure development | 2/10 | 0.046628558 | 2 |
| GO:0006509 | membrane protein ectodomain proteolysis | 1/10 | 0.046628558 | 1 |
| GO:0044060 | regulation of endocrine process | 1/10 | 0.047690225 | 1 |
| GO:0097178 | ruffle assembly | 1/10 | 0.047690225 | 1 |
| GO:0006775 | fat-soluble vitamin metabolic process | 1/10 | 0.048201993 | 1 |
| GO:0007520 | myoblast fusion | 1/10 | 0.048201993 | 1 |
| GO:0052372 | modulation by symbiont of entry into host | 1/10 | 0.048201993 | 1 |
| GO:0032309 | icosanoid secretion | 1/10 | 0.048702501 | 1 |
| GO:0035987 | endodermal cell differentiation | 1/10 | 0.048702501 | 1 |
| GO:0003012 | muscle system process | 2/10 | 0.048702501 | 2 |
| GO:0014075 | response to amine | 1/10 | 0.049189542 | 1 |
| GO:0030225 | macrophage differentiation | 1/10 | 0.049189542 | 1 |
| GO:0060986 | endocrine hormone secretion | 1/10 | 0.049189542 | 1 |
| GO:0051346 | negative regulation of hydrolase activity | 2/10 | 0.049225922 | 2 |
| GO:1900274 | regulation of phospholipase C activity | 1/10 | 0.049666123 | 1 |

Supplementary Table S5: The GO-MF terms enriched by target genes.

| ID | Description | GeneRatio | p.adjust | Count |
| --- | --- | --- | --- | --- |
| GO:0008009 | chemokine activity | 3/10 | 8.97E-05 | 3 |
| GO:0005125 | cytokine activity | 4/10 | 8.97E-05 | 4 |
| GO:0042379 | chemokine receptor binding | 3/10 | 8.97E-05 | 3 |
| GO:0005126 | cytokine receptor binding | 4/10 | 9.79E-05 | 4 |
| GO:0004222 | metalloendopeptidase activity | 3/10 | 0.000198494 | 3 |
| GO:0045236 | CXCR chemokine receptor binding | 2/10 | 0.000291668 | 2 |
| GO:0004252 | serine-type endopeptidase activity | 3/10 | 0.000450283 | 3 |
| GO:0048018 | receptor ligand activity | 4/10 | 0.000450283 | 4 |
| GO:0030546 | signaling receptor activator activity | 4/10 | 0.000450283 | 4 |
| GO:0008236 | serine-type peptidase activity | 3/10 | 0.000452189 | 3 |
| GO:0008237 | metallopeptidase activity | 3/10 | 0.000452189 | 3 |
| GO:0017171 | serine hydrolase activity | 3/10 | 0.000452189 | 3 |
| GO:0001664 | G protein-coupled receptor binding | 3/10 | 0.001471306 | 3 |
| GO:0004175 | endopeptidase activity | 3/10 | 0.004449761 | 3 |
| GO:0005178 | integrin binding | 2/10 | 0.007568557 | 2 |
| GO:0008603 | cAMP-dependent protein kinase regulator activity | 1/10 | 0.014611899 | 1 |
| GO:0043274 | phospholipase binding | 1/10 | 0.019458668 | 1 |
| GO:0070492 | oligosaccharide binding | 1/10 | 0.019458668 | 1 |
| GO:0005149 | interleukin-1 receptor binding | 1/10 | 0.019658677 | 1 |
| GO:0033691 | sialic acid binding | 1/10 | 0.025641419 | 1 |
| GO:0016702 | oxidoreductase activity | 1/10 | 0.027521647 | 1 |
| GO:0016701 | oxidoreductase activity | 1/10 | 0.027521647 | 1 |
| GO:0042056 | chemoattractant activity | 1/10 | 0.037361787 | 1 |
| GO:0048020 | CCR chemokine receptor binding | 1/10 | 0.045370884 | 1 |
| GO:0004601 | peroxidase activity | 1/10 | 0.048130744 | 1 |
| GO:0016684 | oxidoreductase activity | 1/10 | 0.049790777 | 1 |

Supplementary Table S6: The GO-CC terms enriched by target genes.

| ID | Description | GeneRatio | p.adjust | Count |
| --- | --- | --- | --- | --- |
| GO:0045121 | membrane raft | 3/10 | 0.004586843 | 3 |
| GO:0098857 | membrane microdomain | 3/10 | 0.004586843 | 3 |
| GO:0098589 | membrane region | 3/10 | 0.004586843 | 3 |
| GO:0005901 | caveola | 2/10 | 0.004586843 | 2 |
| GO:0044853 | plasma membrane raft | 2/10 | 0.00693311 | 2 |

Supplementary Table S7: Signaling pathway enrichment analysis of the target genes associated with ulcerative colitis

| ID | Description | GeneRatio | p.adjust | Count |
| --- | --- | --- | --- | --- |
| hsa04657 | IL-17 signaling pathway | 8/10 | 8.44E-13 | 8 |
| hsa04668 | TNF signaling pathway | 8/10 | 1.79E-12 | 8 |
| hsa05417 | Lipid and atherosclerosis | 8/10 | 2.44E-10 | 8 |
| hsa05323 | Rheumatoid arthritis | 6/10 | 7.75E-09 | 6 |
| hsa05144 | Malaria | 5/10 | 2.83E-08 | 5 |
| hsa04933 | AGE-RAGE signaling pathway in diabetic complications | 5/10 | 8.17E-07 | 5 |
| hsa05171 | Coronavirus disease - COVID-19 | 6/10 | 1.11E-06 | 6 |
| hsa05418 | Fluid shear stress and atherosclerosis | 5/10 | 3.21E-06 | 5 |
| hsa05164 | Influenza A | 5/10 | 8.24E-06 | 5 |
| hsa04064 | NF-kappa B signaling pathway | 4/10 | 4.00E-05 | 4 |
| hsa05143 | African trypanosomiasis | 3/10 | 7.38E-05 | 3 |
| hsa05219 | Bladder cancer | 3/10 | 9.26E-05 | 3 |
| hsa05163 | Human cytomegalovirus infection | 4/10 | 0.000647028 | 4 |
| hsa04061 | Viral protein interaction with cytokine and cytokine receptor | 3/10 | 0.001139068 | 3 |
| hsa05142 | Chagas disease | 3/10 | 0.001139068 | 3 |
| hsa04620 | Toll-like receptor signaling pathway | 3/10 | 0.001139068 | 3 |
| hsa04060 | Cytokine-cytokine receptor interaction | 4/10 | 0.001410481 | 4 |
| hsa05135 | Yersinia infection | 3/10 | 0.002281236 | 3 |
| hsa04621 | NOD-like receptor signaling pathway | 3/10 | 0.004868641 | 3 |
| hsa04062 | Chemokine signaling pathway | 3/10 | 0.005063075 | 3 |
| hsa05202 | Transcriptional misregulation in cancer | 3/10 | 0.005063075 | 3 |
| hsa05167 | Kaposi sarcoma-associated herpesvirus infection | 3/10 | 0.005063075 | 3 |
| hsa05134 | Legionellosis | 2/10 | 0.007244213 | 2 |
| hsa04623 | Cytosolic DNA-sensing pathway | 2/10 | 0.008461757 | 2 |
| hsa04622 | RIG-I-like receptor signaling pathway | 2/10 | 0.009998708 | 2 |
| hsa05133 | Pertussis | 2/10 | 0.011165403 | 2 |
| hsa05140 | Leishmaniasis | 2/10 | 0.011165403 | 2 |
| hsa05215 | Prostate cancer | 2/10 | 0.01690813 | 2 |
| hsa05146 | Amoebiasis | 2/10 | 0.018001275 | 2 |
| hsa04625 | C-type lectin receptor signaling pathway | 2/10 | 0.018069937 | 2 |
| hsa04670 | Leukocyte transendothelial migration | 2/10 | 0.020891367 | 2 |
| hsa04926 | Relaxin signaling pathway | 2/10 | 0.025686297 | 2 |
| hsa04514 | Cell adhesion molecules | 2/10 | 0.032272965 | 2 |
| hsa04932 | Non-alcoholic fatty liver disease | 2/10 | 0.032272965 | 2 |
| hsa05161 | Hepatitis B | 2/10 | 0.036297574 | 2 |

Supplementary Table S8: Structural information of Hub gene

| Hub targets | PBD ID | Method | Chain | Positions | Resolution |
| --- | --- | --- | --- | --- | --- |
| CXCL8 | 5D14 | X-ray | A | 30-99 | 1.00 Å |
| CCL2 | 3IFD | X-ray | A | 24-99 | 1.90 Å |
| ICAM1 | 1IAM | X-ray | A | 28-212 | 2.10 Å |
| IL1B | 1I1B | X-ray | A | 117-269 | 2.00 Å |
| MMP9 | 1L6J | X-ray | A | 20-444 | 2.50 Å |
| PTGS2 | 5IKR | X-ray | A/B | 19-569 | 2.34 Å |
| MMP3 | 1SLM | X-ray | A | 18-272 | 1.90 Å |
| MMP1 | 1CGE | X-ray | A | 102-269 | 1.90 Å |
| CXCL10 | 1O7Z | X-ray | A/B | 22-98 | 1.92 Å |
| SELE | 1ESL | X-ray | A | 22-183 | 2.00 Å |
